# *PEStimate*: predicting offspring disease risk after polygenic embryo screening

**DOI:** 10.1093/bioinformatics/btag308

**Published:** 2026-05-14

**Authors:** Liraz Klausner, Ateret Revital, Todd Lencz, Shai Carmi

**Affiliations:** Braun School of Public Health and Community Medicine, The Hebrew University of Jerusalem, Jerusalem, 9112102, Israel; Braun School of Public Health and Community Medicine, The Hebrew University of Jerusalem, Jerusalem, 9112102, Israel; Institute of Behavioral Science, Feinstein Institutes for Medical Research, Manhasset, NY 11030, United States; Division of Psychiatry Research, Zucker Hillside Hospital, Glen Oaks, NY 11004, United States; Department of Psychiatry, Zucker School of Medicine at Hofstra/Northwell, Hempstead, NY 11549, United States; Department of Molecular MedicineZucker School of Medicine at Hofstra/Northwell, Hempstead, NY 11549, United States; Braun School of Public Health and Community Medicine, The Hebrew University of Jerusalem, Jerusalem, 9112102, Israel

## Abstract

**Motivation:**

Polygenic embryo screening (PES) is a new, controversial technology, whereby human *in vitro* fertilization embryos are screened for their genetic risk of complex, polygenic diseases. PES aims to reduce the disease burden in offspring by prioritizing the selection of low-risk embryos. However, given that polygenic diseases are usually late-onset, PES outcomes must be estimated by epidemiological modeling. The liability threshold model has been previously used to predict outcomes. However, predictions rely on complex sets of equations, some of which require numerical integration or simulation. Further, previous models failed to account for the possibility that the selected embryo will not be born.

**Results:**

Here, we present *PEStimate*, a freely available online app for predicting PES outcomes when screening for a single disease. *PEStimate* predicts the offspring risk with and without PES, as well as generates plots of the risk reduction versus key parameters. Users can adjust the number of available embryos, the live birth rate, the disease prevalence, the accuracy of the genetic risk predictor, the embryo selection method, the genetic risk of parents, and the disease status of parents, siblings, uncles/aunts, and grandparents of the embryos. Our model includes, for the first time, the possibility of embryo implantation failure, showing that risk reductions have been previously overestimated. *PEStimate* provides geneticists, healthcare professionals, patients, and other stakeholders with a necessary tool for examining the impact of PES and weighing its potential benefits against possible personal and societal harms.

**Availability and implementation:**

*PEStimate*: https://polygenicembryo.shinyapps.io/pestimate. Source code: https://github.com/Lirazk/PEStimate.

## 1 Introduction

Evaluation of the genetic composition of *in vitro* fertilization (IVF) embryos, called preimplantation genetic testing, has been used for several decades to avoid pregnancies with embryos carrying pathogenic variants or chromosomal aberrations (Cimadomo *et al.* 2020). Developments in whole-genome amplification, sequencing, and genotyping methods have made it feasible and affordable to generate genome-wide data for embryos (Treff *et al.* 2019b, Murphy *et al.* 2020, Masset *et al.* 2022, Backenroth *et al.* 2023, Handyside *et al.* 2025, Xia *et al.* 2024, Janssen *et al.* 2024, Shuyuan Li *et al.* 2025). Recently, multiple companies started offering IVF patients screening of their embryos for complex, polygenic diseases, including, e.g., coronary artery disease, hypertension, diabetes (types 1 and 2), breast and prostate cancers, inflammatory bowel disease, and schizophrenia (Treff *et al.* 2019a, Treff *et al.* 2020, Kumar *et al.* 2022, Shenglai Li *et al.* 2024, Xu *et al.* 2025, Moore *et al.* 2025). Each of these conditions is influenced by numerous genes and variants, in addition to non-genetic risk factors. The genetic risk for each condition is usually estimated as a polygenic risk score (PRS), a weighted count of the number of risk-associated alleles carried by an individual (Wang *et al.* 2022, Lewis and Vassos 2022). Embryos are then prioritized for transfer to the uterus of the IVF patient by their PRS for one or more conditions.

Polygenic embryo screening (PES) was criticized by multiple professional societies, authors, and clinicians for a lack of clinical utility. Reasons include the generally low predictive accuracy of PRS, coupled with a small number of embryos, the inability to experimentally validate its predicted health gains, the small absolute risk reductions, the lower accuracy in non-European ancestries, and the possibility of changes in PRS accuracy over time (Turley *et al.* 2021, Forzano *et al.* 2022, Polyakov *et al.* 2022, Treff *et al.* 2022, Siermann *et al.* 2023, Capalbo *et al.* 2024, Grebe *et al.* 2024, Barlevy *et al.* 2024). It was also criticized for possible harms to patients, including discarding viable embryos, an unnecessary exposure to IVF, insufficient counseling, choice overload, and possible long-term adverse psychological impact. Finally, ethical and social harms include a slippery slope toward eugenics and selection for traits, increasing stigmatization and discrimination, unequal access and benefits across populations, distraction from non-genetic causes of disease, commercialization and overmedicalization of reproduction, and changing reproductive norms and parent-child relationships (Turley *et al.* 2021, Forzano *et al.* 2022, Polyakov *et al.* 2022, Treff *et al.* 2022, Todd Lencz *et al.* 2022, Griffin and Gordon 2023, Capalbo *et al.* 2024, Siermann *et al.* 2024a, Grebe *et al.* 2024, Forte *et al.* 2025).

Given that diseases screened by PES typically develop only in adolescence or adulthood, the reduction in disease burden in the next generation that may be achieved by PES must be predicted based on epidemiological models. A number of studies used the liability threshold model (see Section 2) (Treff *et al.* 2019a, Lencz *et al.* 2021, Turley *et al.* 2021, Moore *et al.* 2025), finding that PES may lead to significant risk reductions, at least when having many embryos and under ideal conditions, of up to 50% when screening common diseases such as schizophrenia or Crohn’s disease. The risk reduction can also be estimated by comparing disease outcomes between adult siblings by selecting between the siblings as if they were IVF embryos. An analysis of sibling pairs from the UK Biobank predicted large risk reductions even when siblings are prioritized based on their combined genetic risk for multiple diseases (Treff *et al.* 2020, Widen *et al.* 2022).

To facilitate informed decision-making by patients, clinicians, researchers, and policymakers, it is crucial that all stakeholders have access to clear and evidence-based information on PES. However, the model-based predicted risk reductions rely on a series of complex equations that require numerical multiple integration for each setting of interest. Alternatively, risk reductions based on biobank data are limited to the specific conditions documented in the biobank, the currently existing PRS, and a comparison of usually only two siblings.

Risk reduction calculators are currently available only from companies offering PES: Genomic Prediction (“Risk Reduction Calculator” n.d.), Orchid Health (“The Impact of Family History on the Lifetime Prevalence of Disease” n.d.; “Orchid | Embryo Risk Calculator” n.d.), and Herasight (“Herasight | Advanced Genetic Screening” n.d., Moore *et al.* 2025). These calculators also leave multiple gaps. The Genomic Prediction calculator is based on *unrelated* individuals from the UK Biobank. However, given that PRS is more variable among unrelated individuals than it is among sibling embryos, risk reductions are overestimated (Widen *et al.* 2022). Further, the calculator is limited to specific phenotypes, scores, and UK-based European ancestry individuals. The calculator by Orchid is based on pre-computed simulations of the liability threshold model, and it is thus again limited to specific phenotypes and scores. The Orchid calculator permits specification of the disease status of some of the first- and second-degree family members of the embryo but not that of already born children. The Herasight calculator is also limited to pre-computed model simulations of specific phenotypes. It permits specification of parental disease history and ancestry, as well as ancestry- and sex-specific disease prevalence and heritability.

In addition to the above-mentioned gaps, all currently available risk reduction estimates assume that an embryo will be born once transferred. However, even chromosomally intact embryos will often fail to implant or will result in a miscarriage (Cimadomo *et al.* 2023). Thus, it is the number of potential *births* per IVF cycle, rather than the number of *embryos*, which determines the predicted risk reduction. However, the number of births will not be known to IVF patients at the stage of embryo screening. Therefore, more realistic models of PES must take into account the uncertain nature of embryo implantation outcomes.

Here, we present *PEStimate*, an online app for estimating risk reduction for embryo selection for a single disease. *PEStimate* predicts risk reductions based on analytical solutions to the liability threshold model (Lencz *et al.* 2021), and it is, therefore, fast and flexible. The online interface permits users to estimate the risk reduction for any given parameter set and select from a prespecified list of disease parameters, as well as plot graphs of the risk reduction versus key parameters. Further, we incorporate, for the first time, embryo birth parameters into the PES model, demonstrating that risk reductions were previously overestimated. A preliminary version of the app has been mentioned, without additional information, as part of our 2024 review paper (Capalbo *et al.* 2024). Here, we present a much-expanded version of the app, along with complete details on its underlying models, implementation, and user interface. We believe that *PEStimate* will become a valuable tool for researchers, clinicians, patients, and other stakeholders who are interested in evaluating the prospects of PES.

## 2 Methods

The Supplementary Material, available as supplementary data at *Bioinformatics* online provides complete details on our modeling assumptions and the derivation of the risk reduction under each setting. Here, we briefly describe the key components of our models.

### 2.1 The setting

We assume that embryos were generated by IVF patients in a single oocyte-retrieval cycle and that n embryos have developed to the blastocyst stage. The genome of each embryo has been sequenced or genotyped, and all embryos were euploid (i.e., free of chromosomal aberrations). The embryos are then screened for a prespecified disease with prevalence/lifetime risk K. The disease is assumed to be polygenic, with a normally distributed PRS explaining a proportion $r^{2}$ of the variance in disease liability (see below) in the target population. The target population may be of any ancestry, including non-European or admixed. We assume that K and $r^{2}$ are known from published disease-specific studies. It is possible to convert (Lee *et al.* 2012, Pain *et al.* 2022, Moore *et al.* 2025) multiple metrics of PRS accuracy (e.g. the AUC) into $r^{2}$. The value used for $r^{2}$ should represent only the impact of direct, within-family effects, i.e. not incorporating confounding due to, e.g. population structure, assortative mating, or correlation with parental genotypes (Morris *et al.* 2020). We allow users to adjust $r^{2}$ to non-European populations using the reductions reported (relative to people of Northwestern European ancestry in the UK Biobank) in Privé *et al.* (2022) (Fig. 2 therein).

### 2.2 The genetic risk model

We use the liability threshold model (Dempster and Lerner 1950, Falconer 1965), a popular framework for modeling and predicting risk in genetic epidemiology (So *et al.* 2011, Do *et al.* 2012, Weissbrod *et al*. 2018, Hujoel *et al.* 2020, Yuan *et al.* 2020, Pedersen *et al.* 2022, Dybdahl Krebs *et al.* 2024). The model assumes that the binary disease status is determined by an underlying, unobserved, continuous liability and that individuals with a liability above a threshold are affected. In the context of embryo selection, the model can be written, for n embryos (Lencz *et al.* 2021), as


(1) $$y_{i}=s_{i}+g_{i}^{'}+\epsilon_{i};i=1,\ldots ,n$$

In Equation (1), $y_{i}$ is the (total) liability of embryo i for the specific disease screened. Assuming that the liability is distributed as a standard normal variable, and given the disease prevalence K, the disease threshold is $z_{K}=\Phi^{-1}(1-K)$, where Φ(⋅) is the cumulative distribution function of the standard normal distribution.

The liability in Equation (1) is a sum of genetic and non-genetic risk factors. $s_{i}$ is the PRS (or “score”) of embryo i; $g_{i}^{\prime }$ represents any remaining genetic factors not captured by the score; and $\epsilon_{i}$ represents non-genetic, random, or environmental risk factors. All components are assumed to be independent and normally distributed with zero means (see the Supplementary Material, available as supplementary data at *Bioinformatics* online for more details on these assumptions). The variance of $s_{i}$ is denoted as $r^{2}$, which is also the proportion of variance in liability explained by the PRS. It is used as a measure of the PRS accuracy. The variance of $g_{i}^{\prime }$ is $h^{2}-r^{2}$, such that the overall variance of the genetic risk factors is equal to the heritability $h^{2}$. The variance of $\epsilon_{i}$ is the remaining $1-h^{2}$.

We assume that non-genetic risk factors are independent between family members, and similarly for the genetic risk factors of the parents. In contrast, all genetic factors have a correlation of 1/2 between first-degree genetic relatives (e.g. pairs of embryos or an embryo and a parent). This results in the following form of the liability of each embryo (Lencz *et al.* 2021),


(2) $$y_{i}=c+x_{i}+w+v_{i}+\epsilon_{i}.$$

In Equation (2), the PRS is broken down into $s_{i}=c+x_{i}$, where c is *shared* between all embryos, while $x_{i}∼N(0,r^{2}/2)$ is an *embryo-specific* component. It can be shown (Lencz *et al.* 2021) that $c=(s_{m}+s_{f})/2$, where $s_{m}$ and $s_{f}$ are the maternal and paternal scores, respectively, i.e. c is the mean parental PRS. Similarly, $g_{i}^{\prime }=w+v_{i}$, where $w=(g_{m}^{\prime }+g_{f}^{\prime })/2$ is the mean parental non-PRS genetic component and $v_{i}∼N(0,(h^{2}-r^{2})/2)$ is embryo-specific. Equation (2) also holds for any previously born sibling of the embryos.

### 2.3 Family information

The baseline model for the risk of the selected embryo assumes that the parents are random individuals from the population. It is also possible to specify the PRS of the parents and the disease status of the parents and the following additional relatives of the embryos: grandparents, uncles/aunts, and siblings (i.e. previously born children). The distribution of the genetic liabilities of the family members is then conditioned upon their disease status.

### 2.4 Embryo selection

We consider two embryo selection strategies (Lencz *et al.* 2021). (i) *Lowest-risk prioritization*: selection of the embryo with the lowest PRS (equivalently the lowest $x_{i}$, for i=1,…,n). (ii) High-risk exclusion: exclusion of all embryos with PRS above a cutoff (based on a PRS quantile q). If all embryos are high risk, a random embryo is selected. It is then assumed that the selected embryo is transferred to the uterus of the patient for the purpose of initiating a pregnancy.

### 2.5 The number of embryos/births

If an embryo is transferred but not born, its PRS is irrelevant for embryo screening. Therefore, the only parameter relevant for risk reduction in a given IVF cycle is the number of births. We derive the risk of the selected embryo under the following three models.

1. There are *n* embryos, and each embryo transferred is born. This model is unrealistic, but it provides a useful baseline. In this model, the number of births is fixed at *n*.
2. There are *n* embryos, and each embryo transferred is born with probability *p*. In this model, the number of births is binomial with parameters (*n*,*p*).
3. The number of embryos is Poisson distributed with mean *n*, and each embryo transferred is born with probability *p*. In this model, the number of births is Poisson with mean *np* (see Section 5, available as supplementary data at *Bioinformatics* online).

For models (2) and (3), we assume that at least one birth was achieved, or otherwise the risk reduction is undefined. Equivalently, if the IVF cycle resulted in no live births, it is assumed that additional cycles were performed until at least one birth was achieved. These models are implemented in the app only for the lowest-risk prioritization strategy.

### 2.6 Estimating the risk reductions

Our goal is to estimate the risk of the selected embryo, i.e., the probability of the embryo being affected as an adult. Under each of the settings described above, we analytically derive the risk of the selected embryo in the Supplementary Material, available as supplementary data at *Bioinformatics* online or use the published result (Lencz *et al.* 2021). The risk is always given as a single or a multiple integral. The Supplementary Material, available as supplementary data at *Bioinformatics* online describes how *PEStimate* solves these integrals numerically or using a Monte Carlo approach. We implemented all numerical methods in R (version 4.5.1) and the user interface in R Shiny (version 1.10.0).

We report the following risk reduction metrics. The absolute risk reduction is defined as


(3) ARR=K-P(disease),

where K is the disease prevalence and P(disease) is the risk of the selected embryo. We slightly abuse notation here to use K to denote both the risk of a random individual/embryo from the population and the baseline risk of an embryo in case family information is given.

The relative risk reduction is defined as


(4) $$\mathrm{RRR}=\frac{K-P(\mathrm{disease})}{K}.$$

The number of patients that would need to be screened in order to avoid a single disease case is computed as NNS=1/ARR.

## 3 Results

### 3.1 The impact of modeling the possibility of embryo transfer failure

All previous models for PES outcomes (Treff *et al.* 2019a, Turley *et al.* 2021, Lencz *et al.* 2021, Moore *et al.* 2025) reported risk reductions versus the number of (euploid) embryos being screened, implicitly assuming that the transferred embryo will be born. However, given that many euploid embryo transfers fail to result in live birth, the availability of a given number of embryos does not guarantee the predicted risk reductions. Thus, the number of embryos must be replaced with the number of *potential births* from all embryos generated in the egg retrieval cycle. However, for any given number of embryos, the number of births is not known prior to embryo transfer, and it is better modeled as a random variable.

A naive approach to modeling risk reduction may be setting the number of births to the *expected (mean) number of births* given the embryo count or other parental parameters. However, in the Supplementary Material, available as supplementary data at *Bioinformatics* online, we prove that


(5) $$\mathrm{RRR}(E(N))\geq E_{N}(\mathrm{RRR}(N)).$$

In Equation (5), N is the number of births and RRR is the relative risk reduction (see Section 2; Equation 4). The inequality demonstrates that using the mean number of births, E(N), and then predicting the corresponding relative risk reduction (i.e. RRR(E(N))) always overestimates the risk reduction compared to properly averaging the risk reduction over all possible numbers of births ($E_{N}(\mathrm{RRR}(N))$. Thus, the naive approach would lead to over-optimistic predictions.

To address this problem, we propose two new models for the number of births. In the first, the number of embryos is given, and each embryo is born with a certain live birth probability (the “binomial” model; see Section 2). In the second, the number of embryos is also random (the “Poisson” model).

**Figure 1:**
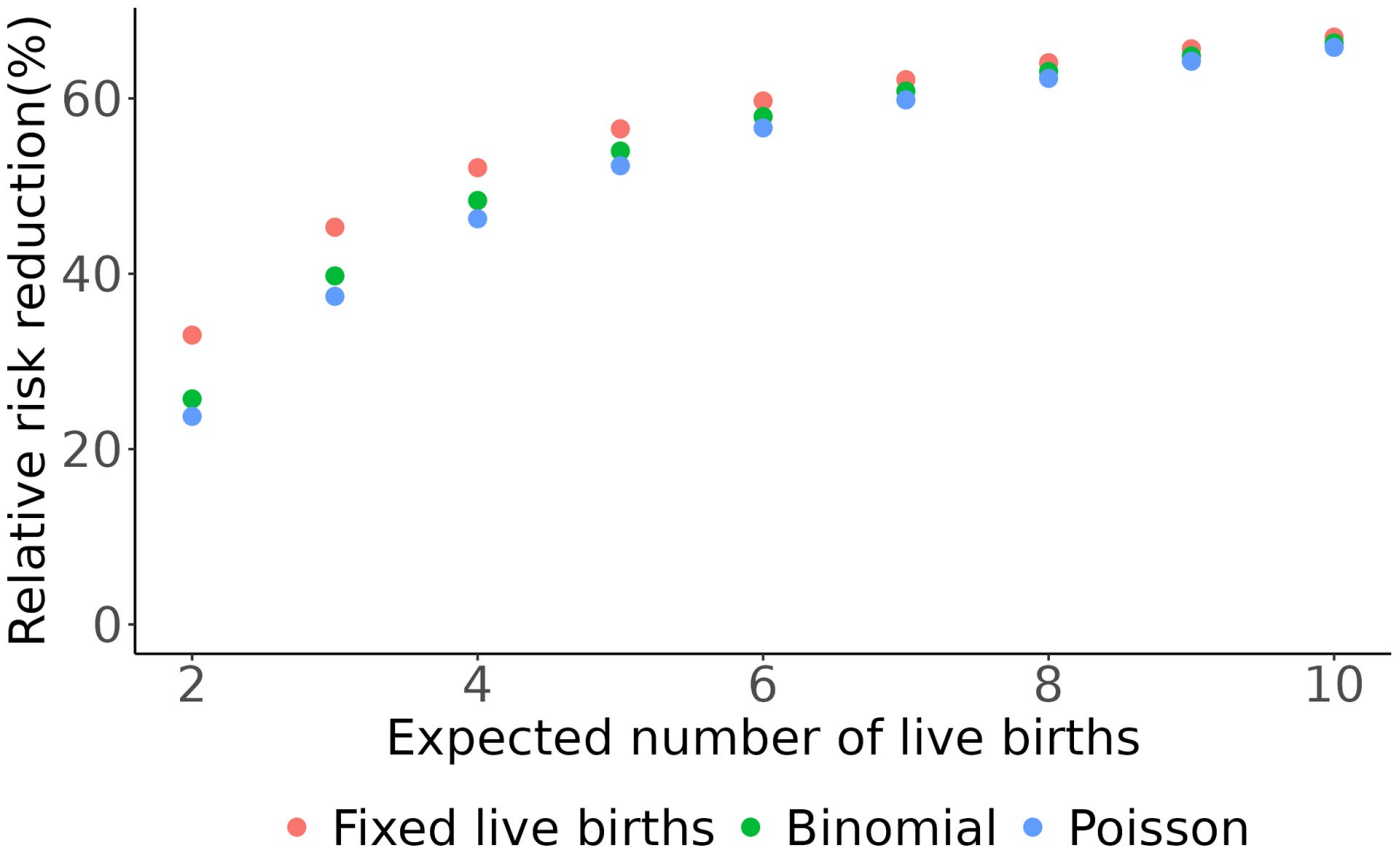
A comparison of relative risk reductions predicted by three models for the number of births. We consider the lowest-risk prioritization selection strategy and assume at least one birth. We set the live birth rate to p=0.4, the prevalence to K=1%, and the PRS accuracy to $r^{2}=0.1$. We set the other model parameters such that the mean number of births is the same across all three models (see the Supplementary Material, available as supplementary data at *Bioinformatics* online).

We compare predictions from the three models—the binomial model, the Poisson model, and fixing the number of births to its mean—in Fig. 1. To achieve a fair comparison, we selected model parameters in a way that the mean number of births is the same across models (see the Supplementary Material, available as supplementary data at *Bioinformatics* online). As predicted by Equation (5), using a fixed number of births indeed overestimates the risk reduction. Overestimation is substantial up to ≈4–5 average births per cycle. The predictions of the two models with a random number of births are numerically similar. These observations are consistent across various values for the prevalence and the PRS accuracy.

### 3.2 An online risk reduction estimation app

We implemented an online graphical user interface for the estimation of risk reductions as an R Shiny app. The app has three pages.

The first is an “About” page, providing a brief description of the app, the embryo selection strategies, the input and output parameters, references, and contact information.

The two other pages are “Calculator” and “Plot”. On the first visit to either of those pages, the user is presented with a pop-up window with “Important notes.” Most of these notes inform the user on the model’s assumptions, as described in the previous sections. The last note indicates that PES has been associated with ethical, social, and legal problems. The user must confirm reading and understanding the notes and that the app is intended for research purposes only.

**Figure 2:**
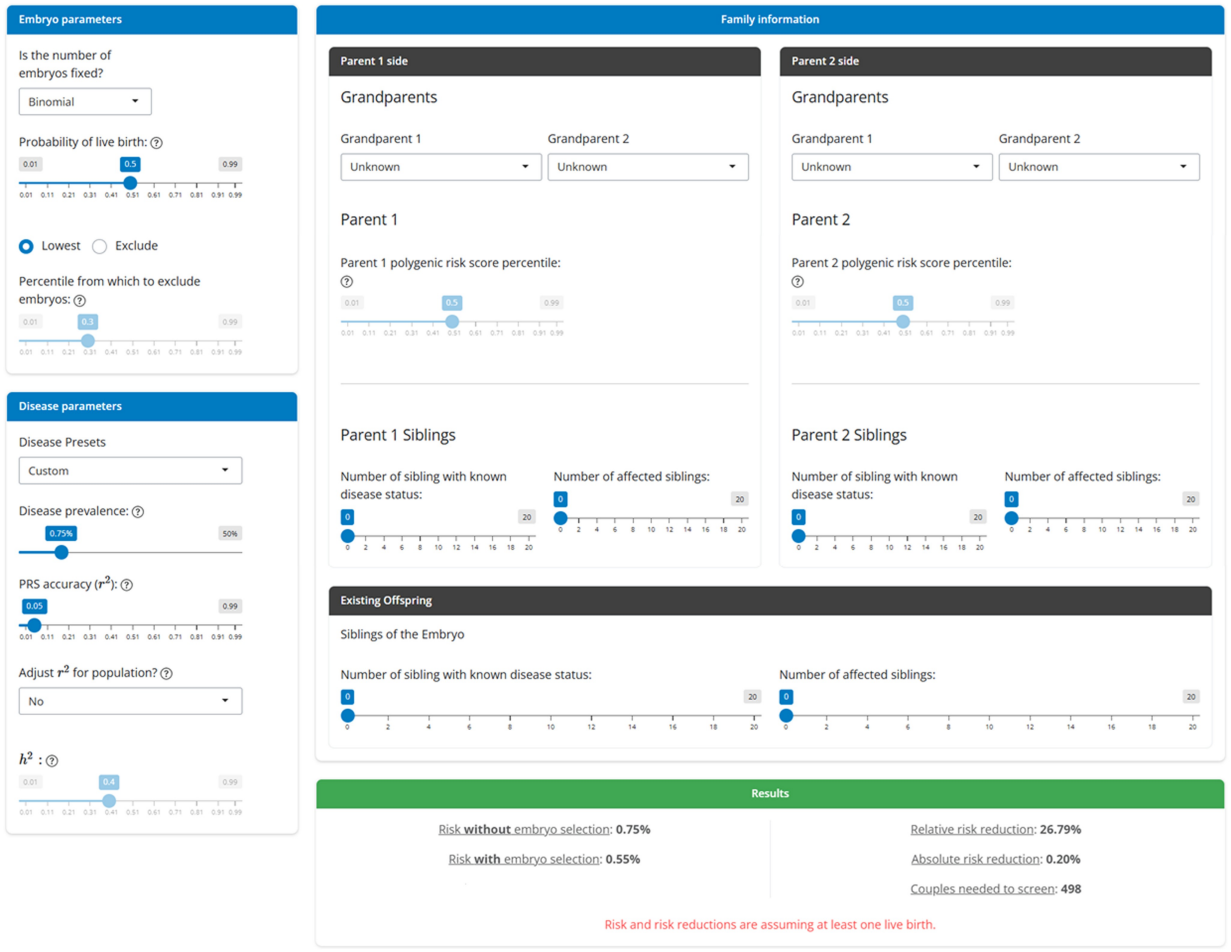
A screenshot of *PEStimate’*s Calculator page. Note that the heritability ($h^{2}$) is only required if the disease status of family members is provided.

The “Calculator” page (Fig. 2) allows users to input all relevant parameters and examine the risk reductions. Most input parameters have an accompanying tooltip. The input parameters are organized in three panels.

The “Embryo parameters” panel first requires users to select a model for the number of embryos/births from among the three models described in Section 2. For a fixed number of births, the user needs to specify the number. For the binomial number of births, the user needs to specify the number of (euploid) embryos and the birth rate per embryo transfer. For the Poisson model, the user needs to specify the *mean* number of embryos and the birth rate. Finally, the user needs to specify the embryo selection strategy (lowest-risk prioritization or high-risk exclusion; see Section 2).

The “Disease parameters” panel specifies the disease prevalence (or the probability that a random individual is affected) and the accuracy of the PRS ($r^{2}$, the proportion of variance in liability explained by the PRS; see Section 2). If the high-risk exclusion strategy is selected, the user must also specify a PRS percentile cutoff. If the disease status of family members is provided (see next), the user must specify the heritability $h^{2}$ (the proportion of variance in liability explained by genetic risk factors; see Section 2; $r^{2}\leq h^{2}\leq 1$). These parameters should be obtained from the relevant literature or in-house data. We provide parameters extracted from the literature (mostly for European ancestry populations) for 17 prespecified diseases (Table 1, available as supplementary data at *Bioinformatics* online). Finally, users can automatically adjust $r^{2}$ to a number of populations of non-Northwestern European ancestry.

The final panel provides information on family members. For each parent, users can input the parental PRS and the disease status of the parent, the parents of the parent (the grandparents of the embryos), and any number of siblings of the parent (uncles/aunts of the embryos). Users can also input the disease status of any number of previously born children (siblings of the embryos). Users can also specify the disease status of these relatives as “unknown.”

The output of the model (rectangle under the green bar at the bottom of Fig. 2) provides the following information. (i) The risk for the future child *without* embryo screening. This is equal to the disease prevalence, except when family information is provided, in which case the risk is based on the given family parameters. (ii) The risk *with* embryo screening. (iii) The relative risk reduction; (iv) the absolute risk reduction; and (v) the number of patients that need to be screened in order to avoid a single disease case (see Section 2). In case the user has provided the disease status of family members, the output (which is based on Monte Carlo sampling) also includes standard deviations.

**Figure 3:**
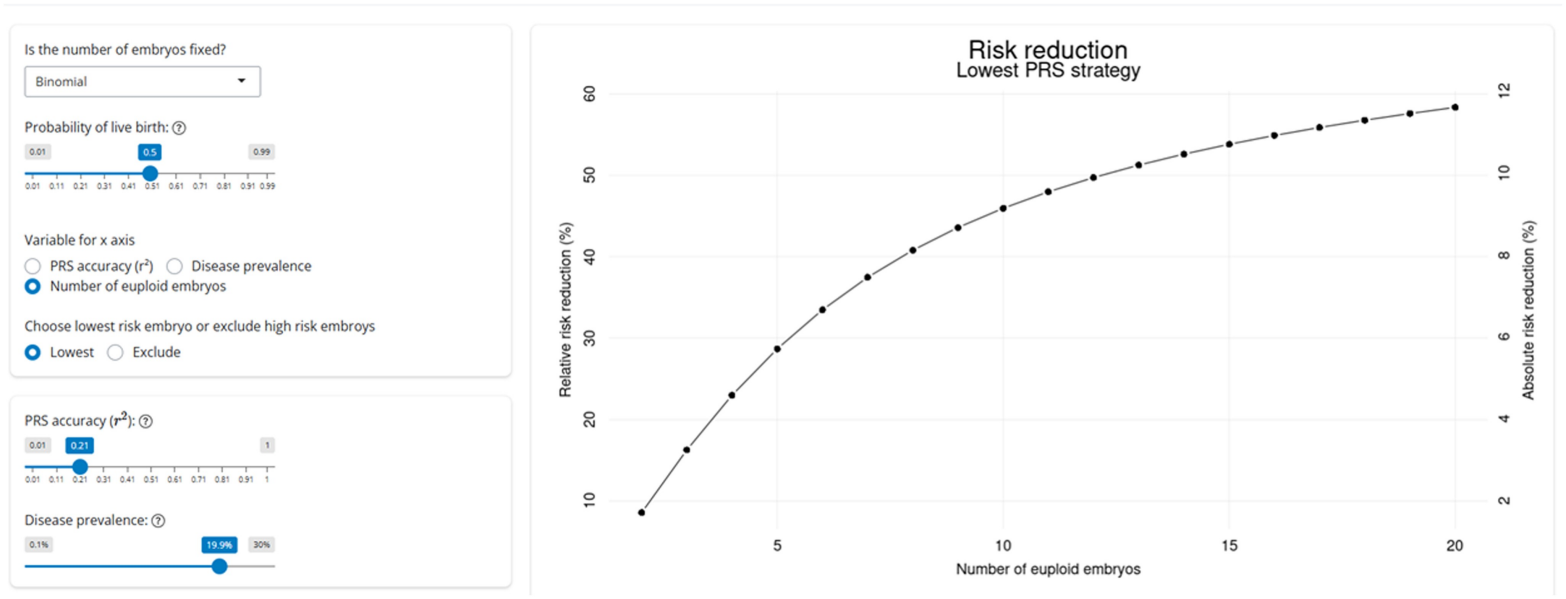
A screenshot of *PEStimate’*s Plot page. The parameters correspond to Case 4 in the Examples subsection.

The “Plot” page (Fig. 3) allows users to plot the relative and absolute risk reductions versus some of the model’s parameters. The model for the number of embryos/births is specified as on the Calculator page. Users can then plot the risk reduction against each of the following parameters: (i) The number of births/embryos (depending on the model). (ii) The disease prevalence. (iii) The PRS accuracy ($r^{2}$). (iv) For the high-risk exclusion strategy, the exclusion cutoff (as a PRS percentile). To limit the complexity of the Plot page, it does not permit specification of family information.

### 3.3 Examples

*Case 1*. One spouse in a couple is affected with schizophrenia, and the other is unaffected. The couple is theoretically fertile, but they have no children yet. What is the schizophrenia risk reduction that can be achieved for their future child by selecting the lowest-risk embryo? We set the prevalence to K=0.87% (Perälä *et al.* 2007), the proportion of variance explained by the PRS to $r^{2}=0.07$ (Legge *et al.* 2024), and the heritability to $h^{2}=0.81$ (Polubriaginof *et al.* 2018). We assume that the couple will have a random (Poisson) number of euploid embryos with mean 4 (Labarta *et al.* 2017, Rodríguez-Varela *et al.* 2024), and we set the birth rate per euploid embryo transfer to 0.5 (Cimadomo *et al.* 2023, “All SART Member Clinics - 2022 Retrieval and Transfer Tables” n.d.). The risk of the future child decreases from 7.95% without PES to 6.43% with PES.

*Case 2*. A couple already has one child who is affected by type 1 diabetes. The parents are unaffected and fertile. The parents were tested, and their paternal and maternal PRS percentiles were at the top 5th and 15th, respectively. They already went through IVF and generated five euploid embryos. What is the type 1 diabetes risk reduction that can be achieved for their future child by selecting the lowest-risk embryo? We set the prevalence to K=0.15% (Gong *et al.* 2025), the PRS accuracy to $r^{2}=0.25\;$(Lee *et al.* 2012, Deutsch *et al.* 2025), the heritability to $h^{2}=0.83\;$(Wei *et al.* 2025), and the birth rate to 0.5. The risk of the future child decreases from 4.45% without PES to 2.24% with PES.

*Case 3*. A couple suffering from infertility went through IVF and generated a number of embryos that would lead to two live births. They have no children yet. They are interested in guaranteeing that their future child is not at high risk for coronary artery disease (for which their own disease status is yet unknown), and thus they would like to exclude all embryos in the top 10% of genetic risk. We set the prevalence to K=8.3% (Truong *et al.* 2024) and the PRS accuracy to $r^{2}=0.05\;$(Truong *et al.* 2024). In this setting, the risk of the future child decreases from 8.3% without PES to 7.91% with PES.

*Case 4*. A healthy, theoretically fertile, yet childless couple is planning to go through IVF to reduce their future child’s risk for type 2 diabetes. They were not genetically tested, and their disease status is yet unknown. They do not know how many euploid embryos they would need to have to achieve a given risk reduction. We set the prevalence to K=19.9% (Moore *et al.* 2025), the PRS accuracy to $r^{2}=0.21$(Moore *et al.* 2025), and the birth rate to 0.5. The risk reduction (relative and absolute) is shown in Fig. 3 as a function of the number of embryos.

## 4 Discussion

PES is an emerging assisted reproductive technology with a rapidly evolving landscape and massive public interest (Dwoskin and Torbati 2025, Dockser Marcus 2025, Sussman 2025). It adds a highly contentious dimension to reproductive decision-making, raising significant ethical, clinical, and social concerns. Further, attitudes toward PES diverge between healthcare professionals, who are reluctant to offer the screen due to perceived low clinical utility and potential for harm, and members of the general public, who show both approval of PES and self-interest (Capalbo *et al.* 2024, Barlevy *et al.* 2024, Furrer *et al.* 2024, Siermann *et al.* 2024b). In this context, it is crucial to provide all stakeholders with an accessible tool for evaluating the potential utility of PES. *PEStimate* addresses this need by allowing users to examine PES outcomes under a wide array of parameters and models.

There are many limitations to our modeling framework, most of them discussed previously (Lencz *et al.* 2021). Briefly, the model relies on the assumption that disease status is truly binary (affected/unaffected), that each disease has an underlying continuous liability, that only individuals with liability above a threshold are affected (Gusev 2025), and that all liability components are normally distributed. Deviations from normality are expected, particularly for conditions with common high-impact pathogenic variants, such as Alzheimer’s disease or breast cancer, and the utility of PES for carriers requires a separate framework (Todd Lencz *et al.* 2026). Additional assumptions include a correlation of 0.5 between the genetic liabilities of first-degree relatives, independence between the non-genetic risk factors of family members, independence between genetic and non-genetic risk factors, and absence of gene-gene or gene–environment interactions. A shared environment between siblings is expected for some conditions (Lakhani *et al.* 2019), but this will only affect our estimates when conditioning on the disease status of an already born child, increasing the baseline risk of the embryos when that child is affected (Section 6, available as supplementary data at *Bioinformatics* online). Gene–environment correlations are also sometimes expected (Abdellaoui *et al.* 2022); risk reductions will increase for positive correlations (Section 6, available as supplementary data at *Bioinformatics* online). In conclusion, results from our model should be considered a crude approximation that is only valid under our assumptions. Nevertheless, the model’s predictions agree closely with simulations (Lencz *et al.* 2021), and our model and app provide important information regarding the impact of the various parameters.

Our approach for modeling a non-deterministic number of births is novel and avoids one of the main pitfalls of existing risk reduction estimators. We use our new model to show that previous risk reduction estimates were inflated, even after accounting for the possibility of transfer failure by using the expected number of births. Naturally, our model also relies on several assumptions. These include a constant live birth rate across families and across all embryos in a given IVF cycle, independence between the birth rate and other parameters, and that at least one birth was achieved. The model with a random number of embryos is limited to a Poisson distribution. However, our analytical framework (Section 5, available as supplementary data at *Bioinformatics* online) can seamlessly incorporate any other distribution of the number of live births. Extending the model and app to additional distributions (e.g., a negative binomial number of embryos) is thus straightforward.

An important limitation of our app is its focus on embryo selection based on a single disease risk. Selecting embryos simultaneously based on their risk for multiple diseases introduces several complications, such as the need to model the correlation between the liability components of pairs of diseases and the explosion in the number of possible selection strategies. While the gain under simultaneous selection for multiple traits can be derived analytically (Karavani *et al.* 2019), no analogous result exists for disease risk, and estimating the risk reduction will require the development of a fast simulation approach, possibly expanding upon our previous method for simulating pairs of diseases (Lencz *et al.* 2021). While we leave modeling of selection against multiple diseases for future work, biobank sibling data may provide a more attainable alternative for estimating risk reductions in the near future.

The main advantage of our app is the ability to perform a fast exploration of the parameter space. This is facilitated by the analytical solutions we derived under most settings and our fast simulation approach for the remaining settings, in contrast to existing calculators that rely on pre-computed simulations. One useful application is examining how PES outcomes are affected by reduced PRS accuracy, e.g., due to ancestry, environmental, or generational differences or by improved PRS accuracy expected in the future.

In conclusion, *PEStimate* allows users to research what approximate risk reductions are expected from PES under various contexts, including different diseases, PRS, embryo counts, and family history. We believe our app will be useful for researchers, healthcare professionals, regulators, and the general public when considering the benefits and harms of the technology.

## Supplementary Material

btag308_Supplementary_Data

## Data Availability

There are no new data associated with this article.
