# Supplementary material for: PEStimate: predicting offspring disease risk after polygenic embryo screening
Source: Bioinformatics. 2026 May 14;42(6):btag308. doi: 10.1093/bioinformatics/btag308 (PMC13255997; doi:10.1093/bioinformatics/btag308)
Supplement: btag308_Supplementary_Data [file btag308_supplementary_data.pdf]

PEStimate: Predicting offspring disease risk after Polygenic Embryo Screening

Supplementary Material

Liraz Klausner, Ateret Revital, Todd Lencz, and Shai Carmi

April 23, 2026

1 Overview

This document provides technical details on the implementation of the polygenic embryo screening (PES) risk reduction calculator. The document is organized as follows. Section 2 presents a mathematical model for PES for a single disease [following up on our previous work (Lencz et al., 2021)]. The model defines the genetic and non-genetic risk factors of the embryo, how they are distributed across the embryos, and how they translate into disease risk. Section 3 describes the integrals that we numerically solve to provide the risk reduction for two selection strategies: lowest risk prioritization and high-risk exclusion. Section 4 describes a Monte Carlo method for estimating the risk reduction when the disease status of family members is known. Section 5 demonstrates how risk reduction can be computed even if the selected embryo is not guaranteed to be born and when the number of embryos is not known in advance. Section 6 studies deviations from the model's assumptions, such as a gene-environment correlation.

2 A model for polygenic embryo screening

Our model is based on Lencz et al., 2021. We assume the availability of n embryos. It is assumed that all embryos are viable, have developed to the blastocyst stage around day-5 post-fertilization, and are euploid (as assessed, e.g., using PGT-A). In this section, we further assume that once the selected embryo is transferred to the uterus of the patient, it will lead to pregnancy and live birth. We relax this assumption in Section 5. We consider embryo selection based on the risk of a single disease.

We model the risk to the embryo using the liability threshold model (Falconer, 1965), under which it is assumed that a disease has an underlying, unobserved, continuous liability, and that the disease emerges if the liability exceeds

a threshold. The liability is assumed to have a standard normal distribution. We write the liability most generally as

$$
\begin{aligned}
y &= g + \epsilon, \\
g &\sim N(0, h^2), \\
\epsilon &\sim N(0, 1 - h^2), \\
y &\sim N(0, 1). \qquad (1)
\end{aligned}
$$

In Eq. (1), g is the additive genetic component of the liability and ϵ is the non-genetic component, which includes the environment and any random contributions. They are assumed to be independent and normally distributed. The variance of g, or h^2, is called the heritability.

The genetic component g is usually unknown. We therefore write

$$
\begin{aligned}
g &= s + g', \\
s &\sim N(0, r^2), \\
g' &\sim N(0, h^2 - r^2). \qquad (2)
\end{aligned}
$$

In Eq. (2), s is the polygenic risk score (PRS), which is computed using variants known to be associated with the disease based on the results of genome-wide association studies. g' is the non-PRS genetic component, assumed to be independent of s. See Section 6.1 for a theoretical justification for the independence assumption. The variance of the PRS is r^2, which is also the proportion of the variance in liability explained by the PRS, and it is thus a measure of the PRS accuracy.

Therefore, for each individual,

$$
y = s + g' + \epsilon. \qquad (3)
$$

For a batch of n embryos, we split the contribution of each genetic component into shared and embryo-specific components. Following Lencz et al., 2021, we write the liability of embryo i, y_i, as a sum of the following independent normal variables,

$$
\begin{aligned}
y_i &= s_i + g'_i + \epsilon_i \\
&= (c + x_i) + (w + v_i) + \epsilon_i, \\
c &\sim N(0, r^2/2), \\
x_i &\sim N(0, r^2/2), \\
w &\sim N(0, (h^2 - r^2)/2), \\
v_i &\sim N(0, (h^2 - r^2)/2), \\
\epsilon_i &\sim N(0, 1 - h^2), \\
y_i &\sim N(0, 1). \qquad (4)
\end{aligned}
$$

In Eq. 4, the PRS of embryo i, s_i, is written as $s_i = c + x_i$. c is the PRS-specific shared genetic component, which is identical between all embryos. It

has variance $r^2/2$, half the variance of the PRS in the population, which is a result of the correlation of 0.5 between any pair of genetic values of full siblings (Wray et al., 2019). x_i is the embryo-specific PRS component, whose variance also equals to $r^2/2$. Together, $s_i = c + x_i$ is normally distributed with variance r^2.

Similarly, the non-PRS genetic component of embryo i, g'_i, is written as $g'_i = w + v_i$, where w is the shared non-PRS genetic component, with variance $(h^2 - r^2)/2$, and v_i is the embryo-specific non-PRS genetic component (with the same variance). Together, $g'_i = w + v_i$ is normally distributed with variance $h^2 - r^2$. Finally, ϵ_i is the embryo-specific non-genetic component, and the total liability y_i is a standard normal variable. All components are assumed to be independent of each other, implying, for example, no shared environment between siblings or gene-environment correlation.

It can be shown that the shared PRS component c is equal to the mean of the maternal and paternal PRSs, s_m and s_f, respectively,

$$c = \frac{s_m + s_f}{2}. \tag{5}$$

Similarly, the shared non-PRS component w is equal to the mean of the maternal and paternal non-PRS genetic components,

$$w = \frac{g'_m + g'_f}{2}. \tag{6}$$

An individual is affected whenever its liability is above the disease threshold. To determine the threshold, we require

$$P(\text{Disease}) = P(y_i > \text{threshold}) = K, \tag{7}$$

where K is the prevalence of the disease in the population. Given that $y_i \sim N(0,1)$, the threshold is $z_K = \Phi^{-1}(1-K)$, where $\Phi(\cdot)$ is the cumulative distribution function of the standard normal variable.

We consider two embryo selection strategies (Lencz et al., 2021).

1. Lowest risk prioritization: Select the embryo j with the minimum PRS for the prespecified disease, i.e., $j = \arg\min_i s_i = \arg\min_i (c + x_i) = \arg\min_i x_i$.

2. High-risk exclusion: We exclude embryos if their PRS for the given disease is above a cutoff, $s_i = c + x_i > z_q r$, where z_q is the PRS upper q-quantile, $z_q = \Phi^{-1}(1-q)$ (multiplied by r to generate the actual PRS cutoff). If one or more embryos remain (i.e., $s_i < z_q r$), one embryo among them is selected at random. If no embryo has PRS below the cutoff, one embryo is selected at random from the entire set of n embryos.

Denote by $P(\text{Disease})$ the risk of the selected embryo, i.e., its probability to be affected with the disease as an adult. We consider a number of risk reduction metrics. The absolute risk reduction, arr, is defined as

$$arr = K - P(\text{disease}). \tag{8}$$

The relative risk reduction, rrr, is defined as

$$rrr = \frac{K - P(\text{disease})}{K}. \tag{9}$$

Finally, the number needed to screen, nns, is the number of IVF patients that would have to screen their embryos to avoid one future disease case. It is computed as $nns = 1/arr$.

See the Glossary for all parameter definitions.

Glossary

K The disease prevalence in the population.

Φ The cumulative distribution function of the standard normal variable.

ϵ The non-genetic component of the liability.

ϕ The probability density function of the standard normal variable.

arr The absolute risk reduction.

c The PRS-specific genetic component of the liability shared between all embryos.

g The (additive) genetic component of the liability (with variance h^2).

g' The non-PRS genetic component of the liability (with variance $h^2 - r^2$).

h^2 The proportion of the variance in the liability due to all additive genetic factors; the *heritability*.

n The number of embryos available for selection.

nns The number of patients that would need to be screened to avoid a single case.

r^2 The proportion of the variance in liability explained by the PRS (the PRS accuracy).

rrr The relative risk reduction.

s The PRS-specific genetic component of the liability (with variance r^2).

v_i The non-PRS genetic component of the liability unique to embryo i.

w The non-PRS genetic component of the liability shared between all embryos.

x_i The PRS-specific genetic component of the liability unique to embryo i.

z_K The upper quantile of the standard normal distribution corresponding to probability K.

z_q The upper quantile of the standard normal distribution corresponding to probability q.

3 Direct integration

As we showed in Lencz et al., 2021, the risk reduction across settings can be expressed as a multiple integral. In all settings except those when disease status is known for any family member, we solve these integrals numerically without resorting to simulations. We describe these integrals here. In Section 4, we address scenarios with a known family disease status.

3.1 Lowest risk prioritization

The disease risk when transferring the embryo with the lowest risk for the given disease is given by Eq. (20) from the appendix of Lencz et al., 2021,

$$P(\text{disease}) = \int_{-\infty}^{\infty} \left[1 - \Phi\left(\frac{z_K - t\sqrt{1 - r^2/2}}{r/\sqrt{2}}\right)\right]^n \phi(t)dt, \tag{10}$$

where $\phi(\cdot)$ is the probability density function of the standard normal variable.

The disease risk conditional on the mean parental score, c, is given by Eq. (23) in Lencz et al., 2021,

$$P(\text{disease} \mid c) = \int_{-\infty}^{\infty} \left[1 - \Phi\left(\frac{z_K - c - t\sqrt{1 - r^2}}{r/\sqrt{2}}\right)\right]^n \phi(t)dt \tag{11}$$

We solve these two integrals numerically using R's `integrate` function, as in Lencz et al., 2021, as this approach is fast and sufficiently accurate.

3.2 High-risk exclusion

Given the mean parental score, the disease risk of the selected embryo when excluding high-risk embryos is given by Eq. (29) in Lencz et al., 2021. We also solve it using R's `integrate`. The unconditional disease risk is given by Eq. (31) therein, which we solve as follows. First, we rewrite the (originally discontinuous) integral as

$$P(\text{disease}) = \int_{-\infty}^{\infty} \phi(u) \Bigg\{ f_1(u;n) \int_{-\infty}^{\sqrt{2}z_q - u} \left[1 - \Phi\left(\frac{z_K - (u+t)r/\sqrt{2}}{\sqrt{1-r^2}}\right)\right] \phi(t)dt \tag{12}$$

$$+ f_2(u;n) \int_{\sqrt{2}z_q - u}^{\infty} \left[1 - \Phi\left(\frac{z_K - (u+t)r/\sqrt{2}}{\sqrt{1-r^2}}\right)\right] \phi(t)dt \Bigg\} du,$$

where $f_1(u;n)$ and $f_2(u;n)$ are defined as

$$f_1(u;n) = \frac{1 - \left[1 - \Phi\left(\sqrt{2}z_q - u\right)\right]^n}{\Phi\left(\sqrt{2}z_q - u\right)}$$

$$f_2(u;n) = \left[1 - \Phi\left(\sqrt{2}z_q - u\right)\right]^{n-1}.$$

To proceed, we rewrite the inner integrals of Eq. (12) in a more generic form

$$\int_h^k \phi(y)\Phi(a+by)dy \tag{13}$$

by using the identity $1 - \Phi(-y) = \Phi(y)$. This generic form of the integral is given in D. B. Owen, 1980. To solve the integral , we first show that it can be written in the following form,

$$[\Phi(k) - \Phi(h)] \times \begin{cases} P(X < Y), & \text{if } b > 0 \\ P(X > Y), & \text{if } b < 0 \end{cases} \tag{14}$$

with

$$X \sim N\left(-\frac{a}{b}, \frac{1}{|b|}\right), Y \sim \text{TN}(0,1;h,k), \tag{15}$$

where $\text{TN}(0,1;h,k)$ denotes the truncated standard normal distribution with range (h,k), and X and Y are independent. To prove Eq. (14), we first consider $b > 0$,

$$[\Phi(k) - \Phi(h)]P(X < Y) = [\Phi(k) - \Phi(h)] \int_h^k P(X < y \mid Y = y) f(Y = y)dy$$

$$= [\Phi(k) - \Phi(h)] \int_h^k \frac{\Phi\left(\frac{y + \frac{a}{b}}{\frac{1}{|b|}}\right)}{\Phi(k) - \Phi(h)} \phi(y)dy = \int_h^k \Phi(a+by)\phi(y)dy. \tag{16}$$

When $b < 0$, we instead calculate

$$[\Phi(k) - \Phi(h)]P(X > Y) = [\Phi(k) - \Phi(h)] \int_h^k \frac{1 - \Phi\left(\frac{y + \frac{a}{b}}{\frac{1}{|b|}}\right)}{\Phi(k) - \Phi(h)} \phi(y)dy$$

$$= [\Phi(k) - \Phi(h)] \int_h^k \frac{1 - \Phi(-a - by)}{\Phi(k) - \Phi(h)} \phi(y)dy = \int_h^k \Phi(a+by)\phi(y)dy. \tag{17}$$

Given the structure of Eq. (12), we henceforth assume $b > 0$. To find the probabilities in Eq. (14), we write $Y' \sim N(0, 1)$ and $Z = X - Y'$. The joint distribution of Z and Y' is

$$\begin{pmatrix} Y' \\ Z \end{pmatrix} \sim N\left[\begin{pmatrix} 0 \\ -\frac{a}{b} \end{pmatrix}, \begin{pmatrix} 1 & -1 \\ -1 & 1+\frac{1}{b^2} \end{pmatrix}\right]$$

Back to the probability in Eq. (14), we need to calculate

$$\begin{aligned} &[\Phi(k) - \Phi(h)]P(X < Y) = [\Phi(k) - \Phi(h)]P(Z < 0 \mid h \leq Y' \leq k) \\ &= [\Phi(k) - \Phi(h)]\frac{P(Z < 0, h \leq Y' \leq k)}{P(h \leq Y' \leq k)} = P(Z < 0, h \leq Y' \leq k). \end{aligned} \tag{18}$$

We thus only need the cumulative distribution function (CDF) of a bivariate normal variable, that is

$$P(Z < 0, h \leq Y' \leq k) = P(Z < 0, Y' \leq k) - P(Z < 0, Y' \leq h).$$

We next define a normalized $Z' = (Z + a/b)/\sqrt{1 + 1/b^2}$, or $Z = \sqrt{1 + 1/b^2}Z' - a/b$. The correlation between Z' and Y' is $\text{cor}(Z', Y') = \text{cor}(Z, Y') \equiv \rho = \frac{-1}{\sqrt{1+1/b^2}}$. This gives

$$\begin{aligned} P(Z < 0, Y' \leq h) &= P\left(Z' < \frac{a/b}{\sqrt{1 + 1/b^2}}, Y' \leq h\right) \\ &= \Phi_2\left(\frac{a/b}{\sqrt{1 + 1/b^2}}, h; \rho = -\frac{1}{\sqrt{1 + 1/b^2}}\right) \end{aligned}$$

and similarly for $P(Z < 0, Y' \leq k)$. Above, $\Phi_2(\cdot, \cdot; \rho)$ is the CDF of a bivariate standard normal distribution with correlation ρ. This CDF can be calculated through Owen's T function (Donald B. Owen, 1956), which is defined as

$$T(h, \alpha) = \frac{1}{2\pi}\int_0^\alpha \frac{\exp(-\frac{1}{2}h^2(1 + x^2))}{1 + x^2}dx.$$

Fast and accurate methods exist for computing the T function numerically (OwenQ R package). Given the T function, the bivariate CDF can be computed as

$$\begin{aligned} \Phi_2(x, y; \rho) = 0.5\Phi(x) + 0.5\Phi(y) - T(x, a_x) - T(y, a_y) \\ - \begin{cases} 0 & \text{if } xy > 0 \text{ or } (xy = 0 \text{ and } x + y \geq 0) \\ 0.5 & \text{otherwise} \end{cases} \end{aligned}$$

with

$$\begin{aligned} a_x &= \frac{y}{x\sqrt{1 - \rho^2}} - \frac{\rho}{\sqrt{1 - \rho^2}}, \\ a_y &= \frac{x}{y\sqrt{1 - \rho^2}} - \frac{\rho}{\sqrt{1 - \rho^2}}. \end{aligned}$$

In Eq. (12), the inner integral was calculated using the T function, where some of the terms were simplified when we substituted $x = -\infty$ or $y = \infty$. We computed the outer integral with R's `integrate` function.

4 Conditioning on family history

4.1 Model

The calculator can also incorporate information on the disease status of family members of the embryo. This is done by sampling the underlying genetic and non-genetic liability components of family members from their joint distribution, subject to linear constraints imposed by their disease status. We assume that the non-genetic risk factors are not shared between family members.

It is sometimes difficult to determine whether an individual is unaffected or *not yet* affected. Our model does not include the possibility of censoring. Thus, in settings involving late-onset diseases and relatively young family members, it is better not to condition on the disease status.

Let the vector ℓ contain all relevant random liability components for the family members considered. We limit our attention to parents, grandparents, uncles/aunts, and (living) siblings of the embryos. We do not use information on the sexes of the relatives. We write ℓ as follows,

$$\ell = (\ell_{gp}, \ell_p, \ell_s). \tag{19}$$

In Eq. (19), ℓ_{gp} holds the liability components of the grandparents,

$$\ell_{gp} = (s_{mgm}, g'_{mgm}, \epsilon_{mgm}, s_{mgf}, g'_{mgf}, \epsilon_{mgf}, s_{pgm}, g'_{pgm}, \epsilon_{pgm}, s_{pgf}, g'_{pgf}, \epsilon_{pgf})^T. \tag{20}$$

In Eq. (20), s_j, g'_j, ϵ_j are the liability components, following the notation of Eq. (3), for grandparent j. The grandparents can be the maternal grandmother (mgm), the maternal grandfather (mgf), the paternal grandmother (pgm), and the paternal grandfather (pgf). Next, ℓ_p holds the liability components of the parents and their siblings (uncles/aunts of the embryos),

$$\ell_p = (x_m, v_m, \epsilon_m, x_f, v_f, \epsilon_f, x_{sm_1}, v_{sm_1}, \epsilon_{sm_1}, \dots, x_{sf_1}, v_{sf_1}, \epsilon_{sf_1}, \dots)^T. \tag{21}$$

In Eq. (21), we specify the liability components, following the notation of Eq. (4), for parents of the embryos and their siblings. These relatives include the mother (m), father (f), siblings of the mother ($sm_1, sm_2, \dots$), and siblings of the father ($sf_1, sf_2, \dots$). For each relative j, we only need to specify the relative-specific liability components x_j, v_j, ϵ_j, because the shared components c_j and w_j can be obtained from the average of s and g' of the parents of that relative. Specifically, $c_m = c_{sm_1} = \cdots = (s_{mgf} + s_{mgm})/2$, $w_m = w_{sm_1} = \cdots = (g'_{mgf} + g'_{mgm})/2$, $c_f = c_{sf_1} = \cdots = (s_{pgf} + s_{pgm})/2$, and $w_f = w_{sf_1} = \cdots = (g'_{pgf} + g'_{pgm})/2$. Finally, ℓ_s holds the liability components of living siblings of the embryos (i.e., previously born children of the parents),

$$\ell_s = (x_{s_1}, v_{s_1}, \epsilon_{s_1}, x_{s_2}, v_{s_2}, \epsilon_{s_2}, \dots)^T. \tag{22}$$

In Eq. (22), we again only need to specify the sibling-specific liability components, x_j, v_j, ϵ_j, where j can be any sibling of the embryo $(s_1, s_2, \ldots)$. The shared components c_j and w_j can be obtained based on the liability components of the parents.

Under the model of Section 2, ℓ follows a multivariate normal distribution, $\ell \sim N(\boldsymbol{\mu}, \boldsymbol{\Sigma})$. Before conditioning on the disease status of family members, the mean vector is $\boldsymbol{\mu} = \mathbf{0}$. In the covariance matrix, $\boldsymbol{\Sigma}$, the variance terms are r^2, $(h^2 - r^2)$, $\frac{r^2}{2}$, $\frac{h^2-r^2}{2}$, and $1 - h^2$ for each s,g',x,v, and ϵ, respectively. The covariance terms are all zero. This is because ℓ only includes child-specific components for the non-founders of the pedigree (i.e., all relatives of the embryos except the grandparents), and these child-specific components are independent based on our model (i.e., assuming no assortative mating, shared environments, gene-environment correlation, etc.).

4.2 Constraints due to known disease status of family members

The liability y_j of a family member j is a linear combination of the components of ℓ. Specifically, for the grandparents, $y = s+g'+\epsilon$. For all other family members, $y = \frac{s_m+s_f}{2} + x + \frac{g'_m+g'_f}{2} + v + \epsilon$, with their specific parental s_m, s_f, g'_f, g'_m. For example, for the embryo's mother and her siblings (aunts/uncles of the embryos), $y = \frac{s_{mgm}+s_{mgf}}{2} + x + \frac{g'_{mgm}+g'_{mgf}}{2} + v + \epsilon$. The disease status of family members impose constraints on their liability,

- If individual j is affected: $y_j \geq z_K$.
- If individual j is unaffected: $y_j < z_K$, which is equivalent to $-y_j > -z_K$.

The constraints for all individuals with a known disease status can be combined into a matrix inequality $\mathbf{G}\ell \geq \mathbf{r}$, where each row of $\mathbf{G}$ defines the linear combination for one individual's liability (negated for unaffected individuals), and $\mathbf{r}$ contains the corresponding thresholds (z_K or $-z_K$). For example, if sibling 1 of the embryos is affected, $y_{s_1} > z_K$, which means that

$$\frac{1}{2}s_m + \frac{1}{2}g'_m + \frac{1}{2}s_f + \frac{1}{2}g'_f + x_{s_1} + v_{s_1} + \epsilon_{s_1} > z_K. \tag{23}$$

Given that s_m, s_f, g'_m, g'_f are not explicitly in ℓ, we can rewrite the preceding equation as

$$\begin{aligned} &\frac{1}{2}\left(\frac{s_{mgm}+s_{mgf}}{2} + x_m\right) + \frac{1}{2}\left(\frac{g'_{mgm}+g'_{mgf}}{2} + v_m\right) + \\ &\frac{1}{2}\left(\frac{s_{pgm}+s_{pgf}}{2} + x_f\right) + \frac{1}{2}\left(\frac{g'_{pgm}+g'_{pgf}}{2} + v_f\right) + \\ &+ x_{s_1} + v_{s_1} + \epsilon_{s_1} > z_K. \end{aligned} \tag{24}$$

Figure 1: Sampling $\ell \sim N(\boldsymbol{\mu}, \boldsymbol{\Sigma})$ subject to $\mathbf{G}\ell \geq \mathbf{r}$

Input: Mean $\boldsymbol{\mu}$, Covariance $\boldsymbol{\Sigma}$, Constraint matrix $\mathbf{G}$, Constraint vector $\mathbf{r}$.
Output: A sample ℓ satisfying the constraints.

1. **Sample the constrained variables:** Draw a sample $\mathbf{y}' = \mathbf{G}\ell \sim N(\mathbf{G}\boldsymbol{\mu}, \mathbf{G}\boldsymbol{\Sigma}\mathbf{G}^T)$ such that $\mathbf{y}' \geq \mathbf{r}$. *(Note: This step requires a specialized multivariate truncated normal sampler.)*
2. **Sample an unconditional base:** Draw an independent sample $\mathbf{z}$ from the original, *unconditional* distribution: $\mathbf{z} \sim N(\boldsymbol{\mu}, \boldsymbol{\Sigma})$.
3. **Compute the conditional sample:** Adjust the unconditional sample $\mathbf{z}$ to match the sampled constrained value $\mathbf{y}'$:

$$\ell = \mathbf{z} + \boldsymbol{\Sigma}\mathbf{G}^T(\mathbf{G}\boldsymbol{\Sigma}\mathbf{G}^T)^{-1}(\mathbf{y}' - \mathbf{G}\mathbf{z})$$

The corresponding row of $\mathbf{G}$ would be

$$(1/4, 1/4, 0, 1/4, 1/4, 0, 1/4, 1/4, 0, 1/4, 1/4, 0, 1/2, 1/2, 0, 1/2, 1/2, 0, ..., 1, 1, 1, ...).$$

When the parental PRS are given (in additional to the disease status of family members), we recompute the mean and variance of all liability components conditional on the known $s_m = (s_{mgm} + s_{mgf})/2 + x_m$, $s_f = (s_{pgm} + s_{pgf})/2 + x_f$ using standard result for conditional multivariate normal distributions. This is required, given that knowledge of the parental PRS affects the distribution of the PRS of the grandparents and the siblings of the parents. We then run the same sampling algorithm (described next) on the conditional distribution.

4.3 Sampling from the constrained distribution

We need to sample ℓ from its distribution conditional on $\mathbf{G}\ell \geq \mathbf{r}$. We use a Monte Carlo approach based on the algorithm of Cong, Chen, and Zhou, 2017. The algorithm is described in Figure 1. It works by decomposing the sampling process: first sample the constrained variable $\mathbf{y}' = \mathbf{G}\ell$ from its valid (truncated) range, and then sample ℓ conditional on the drawn value $\mathbf{y}'$. We implemented sampling from multivariate truncated normal using the `tmvtnorm` package (Wilhelm and G, 2023). Other options for generating samples exist (Li and Ghosh, 2015, Cong, Chen, and Zhou, 2017).

4.4 Estimating the risk of the selected embryo

We run the algorithm of Figure 1 repeatedly to generate K independent samples, $\left\{\ell^{(1)}, \ldots, \ell^{(K)}\right\}$, from the conditional distribution. For each sample $\ell^{(k)}$, we first define the shared (mean parental) components of the embryos, $c^{(k)} = \left(s_m^{(k)} + s_f^{(k)}\right)/2$ and $w^{(k)} = \left(g_m'^{(k)} + g_f'^{(k)}\right)/2$. Given $c^{(k)}$ and $w^{(k)}$, the liability of embryo i is $y_i^{(k)} = c^{(k)} + w^{(k)} + x_i + v_i + \epsilon_i$. The embryo-specific components (x_i, v_i, ϵ_i) are sampled independently from their respective distributions: $N(0, r^2/2)$, $N(0, (h^2 - r^2)/2)$, and $N(0, 1 - h^2)$. We then simulate n such embryos. For the lowest-risk prioritization strategy, we select the embryo with the lowest s_i. For the high-risk exclusion strategy, we randomly select an embryo after excluding embryos with PRS above the exclusion cutoff. We then determine whether the liability of the selected embryo is above the threshold (i.e., whether the embryo is affected).

To reduce the variance of the above method, and given that embryo selection depends only on the PRS s_i, we only sample the embryo-specific PRS x_i. We then analytically compute the risk of the selected embryo by integrating over v_i and ϵ_i. Specifically, given that (for simulation k) $y_i = c + w + x_i + v_i + \epsilon_i$, we set $y_i' = c + w + x_i$ based on the simulated data, such that $y = y' + v_i + e_i$. Given that v_i and ϵ_i are independent, their sum has a normal distribution $N(0, (h^2 - r^2)/2 + 1 - h^2) = N(0, 1 - h^2/2 - r^2/2)$. Thus, $P(\text{Disease}) = P(y_i > z_K) = P(y_i' + v_i + \epsilon_i > z_K) = 1 - \Phi\left(\frac{z_K - y_i'}{\sqrt{1 - h^2/2 - r^2/2}}\right)$.

The risk reduction is then computed based on Eqs. (8) and (9). However, K now designates the family-specific prevalence, or the "baseline" risk, i.e., the risk of a random embryo to be affected given the family information.

5 Risk reduction with a variable number of embryos and births

5.1 Introduction

We have so far assumed that (*i*) the selected embryo is always born; and (*ii*) the number of embryos is given. Therefore, we computed the risk reduction as a function of the (fixed) number of embryos n. However, in practice, (*i*) only half or so of the embryos transferred are born (Cimadomo et al., 2023); and (*ii*) stakeholders may be interested in the *expected* risk reduction when the number of embryos is not known in advance (but rather only its distribution).

In this section, we derive the risk reduction for the case when either the number of embryos or the number of births (or both) are random variables. We only consider the lowest risk prioritization strategy; the case of the high-risk exclusion strategy is analogous but more tedious.

5.2 Why does it matter if the number of births is random?

Recall that the relative risk reduction is defined as $rrr = (K - P(\text{Disease}))/K$ (Eq. (9)), where K is the disease prevalence and $P(\text{Disease})$ is the risk of the selected embryo (as computed in previous sections). More generally, denote the relative risk reduction as a function of the number of embryos as $rrr(n)$. Under our initial assumptions, (i) n is the number of potential (live) births for the given IVF cycle, because all embryos can be born if transferred; and (ii) n is a constant. In reality, these assumptions do not hold.

Naturally, an embryo transferred but not born is irrelevant for risk reduction. Therefore, the risk reduction is determined by the number of births. In the most general setting, the number of births is a random variable, denoted N. Naively, we could replace $rrr(n)$, for a fixed n, with $rrr(E(N))$, where $E(N)$ is the mean number of births.

However, we prove that in the case of the lowest risk prioritization strategy,

$$rrr(E(N)) \geq E_N(rrr(N)). \tag{25}$$

In Eq. (25), $E_N(rrr(N))$ is the mean risk reduction over all possible values of N. Thus, the equation implies that the risk reduction achieved by assuming that all IVF cycles result in the same number of births (the mean $E(N)$) is greater than the risk reduction when it is (more realistically) averaged over all possible values of N. Thus, not taking into account the uncertainty in the number of births overestimates the risk reduction.

To prove Eq. (25), it is sufficient to show that $rrr(n)$ is a concave function of n. We show this in Figure 2. Eq. (25) then immediately follows from Jensen's inequality. In the high-risk exclusion strategy, the relative risk reduction is neither concave nor convex.

5.3 Derivation of the mean risk reduction for a random number of births

Denote by N the random variable of the number of births. Our goal is to find

$$E_N[rrr(N)] = E_N\left(\frac{K - P_N(\text{Disease})}{K}\right) = \frac{K - E_N[P_N(\text{Disease})]}{K}. \tag{26}$$

Thus, we need to find $E_N[P_N(\text{Disease})]$.

Denote the probability mass function of N as $P(N = n)$. For a given number of births N, Eq. (10) provides the risk of the selected embryo,

$$P_N(\text{Disease}) = \int_{-\infty}^{\infty}\left[1 - \Phi\left(\frac{z_K - t\sqrt{1 - r^2/2}}{r/\sqrt{2}}\right)\right]^N \phi(t)dt = \int_{-\infty}^{\infty}[B(t)]^N\phi(t)dt, \tag{27}$$

where we defined $B(t) \equiv 1 - \Phi\left(\frac{z_K - t\sqrt{1-r^2/2)}}{r/\sqrt{2}}\right)$, as in Figure 2. The mean risk

Figure 2: **Concavity of the relative risk reduction**

Denote by $P_n(\text{Disease})$ the probability that the selected embryo (out of n) is affected, as computed in Eq. (10) under the lowest risk prioritization strategy. The relative risk reduction is defined as $rrr(n) = \frac{K - P_n(\text{Disease})}{K}$. To show that $rrr(n)$ is concave in n, it is sufficient to show that $P_n(\text{Disease})$ is convex.
Let $B(t) = 1 - \Phi\left(\frac{z_K - t\sqrt{1-r^2/2}}{r/\sqrt{2}}\right)$. Note $0 < B(t) < 1$. Following Eq. (10), the probability of disease given n embryos is $P_n(\text{Disease}) = \int_{-\infty}^{\infty} [B(t)]^n \phi(t)\, dt$. [When $n = 0$, $P_n(\text{Disease})$ is ill-defined. To obtain $rrr(n = 0) = 0$ (i.e., no risk reduction without births), we can set $P_n(\text{Disease}) = K$. Thus, $P_n(\text{Disease}) = K$ for both $n = 0$ and $n = 1$, and the proof below still holds. Otherwise, we assume $n > 0$.]
We demonstrate that $P_n(\text{Disease})$ is convex in two ways, treating n as continuous.

Approach 1: Second derivative test

$$\begin{aligned}\frac{\mathrm{d}^2 P_n}{\mathrm{d}n^2} &= \frac{\mathrm{d}^2}{\mathrm{d}n^2} \int_{-\infty}^{\infty} [B(t)]^n \phi(t)\, dt \\ &= \int_{-\infty}^{\infty} \frac{\mathrm{d}^2}{\mathrm{d}n^2} [B(t)]^n \phi(t)\, dt \\ &= \int_{-\infty}^{\infty} [B(t)]^n [\ln B(t)]^2 \phi(t)\, dt\end{aligned}$$

Since $[B(t)]^n > 0$, $[\ln B(t)]^2 \geq 0$, and $\phi(t) > 0$, the integrand is non-negative. Thus, $\frac{\mathrm{d}^2 P_n}{\mathrm{d}n^2} \geq 0$, implying that $P_n(\text{Disease})$ is convex.

Approach 2: Definition of convexity Let $n = (1-\alpha)n_1 + \alpha n_2$ for $\alpha \in [0, 1]$. The function $f(x) = a^x$ is convex for $a \in (0, 1)$.

$$\begin{aligned}P_n(\text{Disease}) &= \int_{-\infty}^{\infty} [B(t)]^{(1-\alpha)n_1 + \alpha n_2} \phi(t)\, dt \\ &\leq \int_{-\infty}^{\infty} \left((1-\alpha)[B(t)]^{n_1} + \alpha [B(t)]^{n_2}\right) \phi(t)\, dt \quad \text{(Convexity of } [B(t)]^n\text{)} \\ &= (1-\alpha) P_{n_1}(\text{Disease}) + \alpha P_{n_2}(\text{Disease}).\end{aligned}$$

This directly shows $P_n(\text{Disease})$ is convex. Both approaches confirm the convexity of P_n and thus the concavity of the risk reduction.

reduction is

$$\begin{aligned}E_N\left[P_N(\text{Disease})\right] &= E_N\left[\int_{-\infty}^{\infty} [B(t)]^N \phi(t) dt\right] \\ &= \int_{-\infty}^{\infty} E_N\left[B(t)^N\right] \phi(t) dt \\ &= \int_{-\infty}^{\infty} \phi(t) \sum_{n=0}^{\infty} [B(t)]^n P(N = n) dt \\ &= \int_{-\infty}^{\infty} G_N[B(t)] \phi(t) dt.\end{aligned} \tag{28}$$

In the last step, we used the probability generating function (PGF) $G_N(z) = E_N\left(z^N\right) = \sum_{n=0}^{\infty} z^n P(N = n)$.

Therefore, Eq. (28) provides a recipe for computing the probability that the selected embryo is affected. For any given distribution of the number of births per IVF cycle (N), compute the PGF $G_N(z)$, and then solve Eq. (28) numerically.

However, a problem with Eq. (28) is that it also includes the case $N = 0$. But in reality, if there are no births, the risk reduction is not properly defined. In fact, Eq. (10) is nonsensical for $n = 0$. Therefore, in the following, we condition on $N > 0$, implying that IVF cycles are continuously repeated until at least one child is born.

Given the original distribution of N, $P(N = n)$, the probability mass function of the *conditional* number of births is

$$P(N = n \mid N > 0) = \frac{P(N = n)}{1 - P(N = 0)} = \frac{P(N = n)}{1 - P_0}, \tag{29}$$

where we defined $P_0 \equiv P(N = 0)$. Further, the PGF becomes

$$\begin{aligned} G_{N>0}(z) = E_N(z^n \mid N > 0) &= \sum_{n=1}^{\infty} z^n P(N = n \mid N > 0) \\ &= \sum_{n=1}^{\infty} z^n \frac{P(N = n)}{1 - P_0} = \frac{\sum_{n=1}^{\infty} P(N = n) z^n}{1 - P_0} \\ &= \frac{\sum_{n=0}^{\infty} P(N = n) z^n - P_0}{1 - P_0} = \frac{G_N(z) - P_0}{1 - P_0} \end{aligned} \tag{30}$$

Going back to Eq. (28), we substitute Eq. (30).

$$\begin{aligned} E_N\left[P_N(\text{Disease}) \mid N > 0\right] &= \int_{-\infty}^{\infty} G_{N>0}[B(t)]\phi(t)dt \\ &= \int_{-\infty}^{\infty} \frac{G_N[B(t)] - P_0}{1 - P_0}\phi(t)dt. \end{aligned} \tag{31}$$

We have thus obtained an integral expression for the risk of the selected embryo, and thus, via Eq. (26), to the mean relative risk reduction $E_N[rrr(N) \mid N > 0]$. Eq. (31) depends on $G_N(z)$, the PGF of the distribution of N. The next step is to consider specific distributions and obtain $E_N[rrr(N) \mid N > 0]$ for each case.

5.4 Case 1: A fixed number of embryos and a binomial number of births

Suppose that the number of (euploid) embryos n_0 is known. For example, this is the case for IVF patients in a given IVF cycle. We then assume that each transferred embryo has an identical probability p to be born. Thus, the number of births is binomial, $N \sim \text{Bin}(n_0, p)$. Note that we do not condition on $N > 0$

(i.e., that at least one embryo was born), since this conditioning is accounted for in Eq. (31).

To use Eq. (31), we substitute the PGF of a binomial random variable, $G_N(z) = (1-p+pz)^{n_0}$. Further, $P_0 = (1-p)^{n_0}$. Thus, for a fixed number of embryos and a binomial number of births,

$$E_N[P(\text{Disease}) \mid N>0) = \int_{-\infty}^{\infty} \frac{[1-p+pB(t)]^{n_0} - (1-p)^{n_0}}{1-(1-p)^{n_0}} \phi(t)dt \tag{32}$$

We solve Eq. (32) numerically using R's `integrate`.

5.5 Case 2: A Poisson number of embryos and a binomial number of births

The first case assumed that the number of embryos is fixed, which corresponds to the setting of patients with a specific number of euploid embryos. To obtain the mean risk reduction across the population, it is necessary to take into account the randomness in the number of (euploid) embryos.

In the following, we assume a model whereby the number of euploid embryos has a Poisson distribution with mean λ, and then, every transferred embryo is born with probability p. We start with the following lemma.

Lemma: Poisson followed by Binomial Let

$$\begin{aligned} N_1 &\sim \text{Poisson}(\lambda) \\ N_2 \mid N_1 &\sim \text{Bin}(N_1, p) \end{aligned} \tag{33}$$

Then $N_2 \sim \text{Poisson}(\lambda p)$.

Proof: The PGF of a Poisson(λ) random variable is $G_1(z) = e^{\lambda(z-1)}$. The PGF of a binomial variable Bin(N_1, p) is $(1-p+pz)^{N_1}$. Thus,

$$\begin{aligned} G_2(z) &= E_{N_2}\left(z^{N_2}\right) = E_{N_1}\left[E_{N_2}\left(z^{N_2} \mid N_1\right)\right] = E_{N_1}\left[(1-p+pz)^{N_1}\right] \\ &= G_1(1-p+pz) = e^{\lambda[(1-p+pz)-1]} \\ &= e^{\lambda(-p+pz)} = e^{\lambda p(z-1)}, \end{aligned} \tag{34}$$

which is the PGF of a Poisson(λp) distribution. ■

To compute the probability that the selected embryo will be affected, we can substitute Eq. (34) (i.e., $G_N(z) = e^{\lambda p(z-1)}$ into Eq. (31). Further, $P_0 = P(N = 0) = e^{-\lambda p}$. Thus,

$$\begin{aligned} E_N[P(\text{Disease}) \mid N>0) &= \int_{-\infty}^{\infty} \frac{G_N[B(t)] - P_0}{1-P_0} \phi(t)dt \\ &= \int_{-\infty}^{\infty} \frac{e^{\lambda p[B(t)-1]} - e^{-\lambda p}}{1-e^{-\lambda p}} \phi(t)dt. \end{aligned} \tag{35}$$

Using a similar approach, it is possible to derive the risk reduction for additional distributions for the number of embryos, such as the negative binomial. However, for the sake of simplicity, we limited the implementation of *PEStimate* to the Poisson distribution.

5.6 A numerical comparison of the three models

We provided three models for the IVF process:

1. *A fixed number of births.* This model has a single parameter: the number of births N. The risk reduction is based on Eq. (27).

2. *A fixed number of embryos and a binomial number of births.* This model has two parameters: the (fixed) number of embryos n_0 and the birth rate p. The risk reduction is based on Eq. (32).

3. *A Poisson number of embryos and a binomial number of births.* This model has two parameters: the mean number of embryos λ and the birth rate p. The risk reduction is based on Eq. (35).

To allow a fair comparison between the three models, the mean number of births must be the same across all models. Due to conditioning on $N > 0$, the mean number of births in the binomial model is $n_0 p / \left[1-(1-p)^{n_0}\right]$. The mean number of births in the Poisson model is $\lambda p / \left(1-e^{-\lambda p}\right)$. Thus, we require $n_0 p / \left[1-(1-p)^{n_0}\right] = \lambda p / \left(1-e^{-\lambda p}\right) = N$. To achieve this, we vary N in increments of 1 in the range [1,10]. We then set $p = 0.4$ and vary n_0 or λ to achieve equality of the means (accepting the fact that n_0 may be fractional). We further set $r^2 = 0.1$ and $K = 0.01$.

The risk reductions are plotted in Figure 1 of the main text. The predicted risk reduction based on a fixed number of births is higher than that predicted based on a random number of births, as guaranteed by Eq. (25). The two models with a random number of births give similar results.

6 Deviations from model assumptions

In this section, we investigate the impact of deviations from some of the assumptions of the model of Section 2.

6.1 Correlated score and non-score components

In the model of Eq. (2) ($g = s + g'$), we assumed that the score (s) and the non-score (g') genetic components are independent. We provide below some justification for this choice. We follow the model of Turley et al., 2021. Given that g is unknown, it is estimated with noise in genome-wide association studies. Therefore, we can write "raw" PRS as

$$s_{\text{raw}} = g + a, \tag{36}$$

where a is the estimation error, $\text{Cov}\,(g, a) = 0$ and $\text{Var}\,(a) = \sigma^2$. Thus, $\text{Var}\,(s_{\text{raw}}) = h^2 + \sigma^2$, which is not necessarily the proportion of variance in liability explained. To compute the latter, we use the squared correlation between the raw score and the liability. Given that $y = g + \epsilon$ (Eq. (1)),

$$\begin{aligned} r^2 \equiv [\text{cor}\,(y, s_{\text{raw}})]^2 &= \left[\frac{\text{Cov}\,(y, s_{\text{raw}})}{\text{SD}\,(y)\,\text{SD}\,(s_{\text{raw}})}\right]^2 = \left[\frac{\text{Cov}\,(g+\epsilon, g+a)}{1 \cdot \sqrt{h^2+\sigma^2}}\right]^2 \\ &= \left[\frac{\text{Var}\,(g)}{\sqrt{h^2+\sigma^2}}\right]^2 = \frac{h^4}{h^2+\sigma^2}. \end{aligned} \tag{37}$$

Suppose that we hypothetically regress the liability on the score. The intercept will be zero, given that all means are zero. Thus, $\hat{y} = \beta \cdot s_{\text{raw}}$, and the slope, β, is

$$\beta = \frac{\text{Cov}\,(y, s_{\text{raw}})}{\text{Var}\,(s_{\text{raw}})} = \frac{h^2}{h^2+\sigma^2}. \tag{38}$$

We consequently define the scaled score

$$s \equiv \hat{y} = \beta \cdot s_{\text{raw}} = \frac{h^2}{h^2+\sigma^2} \cdot s_{\text{raw}}. \tag{39}$$

The variance of the scaled PRS is equal to the proportion of variance in liability it explains,

$$\text{Var}\,(s) = \text{Var}\left(\frac{h^2}{h^2+\sigma^2} \cdot s_{\text{raw}}\right) = \left(\frac{h^2}{h^2+\sigma^2}\right)^2 \cdot (h^2+\sigma^2) = \frac{h^4}{h^2+\sigma^2} = r^2. \tag{40}$$

Using the scaled PRS, we can rewrite the liability as

$$\begin{aligned} y &= g + \epsilon \\ &= (s_{\text{raw}} - a) + \epsilon \\ &= \beta \cdot s_{\text{raw}} + (1-\beta)s_{\text{raw}} - a + \epsilon \\ &= s + \frac{\sigma^2}{h^2+\sigma^2} s_{\text{raw}} - a + \epsilon \\ &= s + g' + \epsilon, \end{aligned} \tag{41}$$

where

$$g' \equiv \frac{\sigma^2}{h^2+\sigma^2} s_{\text{raw}} - a \tag{42}$$

is the non-score genetic component. Importantly,

$$\begin{aligned}
\text{Cov}\,(s, g') &= \text{Cov}\left(\frac{h^2}{h^2+\sigma^2} s_{\text{raw}}, \frac{\sigma^2}{h^2+\sigma^2} s_{\text{raw}} - a\right) \\
&= \frac{h^2}{h^2+\sigma^2} \frac{\sigma^2}{h^2+\sigma^2} \text{Var}\,(s_{\text{raw}}) - \frac{h^2}{h^2+\sigma^2} \text{Var}\,(a) \\
&= \frac{h^2\sigma^2}{(h^2+\sigma^2)^2}(h^2+\sigma^2) - \frac{h^2\sigma^2}{h^2+\sigma^2} = 0.
\end{aligned} \tag{43}$$

Thus, s and g' are uncorrelated. Assuming that they are jointly normally distributed, s and g' are independent. Given that $s + g' = g$ and that s and g' are uncorrelated, $\text{Var}\,(s + g') = \text{Var}\,(g) = h^2$ and thus $\text{Var}\,(g') = h^2 - r^2$. We have thus restored the model of Eq. (3), using the scaled PRS s and the non-score genetic component g'.

More generally, consider the model $y = s + g' + \epsilon$ as in Section 2, but where s and g' are correlated, $\text{cor}\,(s, g') = \rho$. It is implied that in this model, the variance of the genetic component is $\text{Var}\,(s + g') = r^2 + h^2 + 2\rho r\sqrt{h^2 - r^2}$. Similarly to the above derivation, we hypothetically regress y on s, $\hat{y} = \beta \cdot s$ (there is again no intercept). The slope is

$$\begin{aligned}
\beta &= \frac{\text{Cov}\,(y, s)}{\text{Var}\,(s)} = \frac{\text{Cov}\,(s + g' + \epsilon, s)}{\text{Var}\,(s)} \\
&= \frac{\text{Var}\,(s) + \text{Cov}\,(g', s)}{\text{Var}\,(s)} \\
&= \frac{r^2 + \rho r\sqrt{h^2 - r^2}}{r^2} = 1 + \frac{\rho\sqrt{h^2 - r^2}}{r}.
\end{aligned} \tag{44}$$

We can then define $\tilde{s} = \beta \cdot s$ and write the liability as

$$\begin{aligned}
y &= \tilde{s} + (s - \tilde{s}) + g' + \epsilon \\
&= \tilde{s} + \tilde{g} + \epsilon,
\end{aligned} \tag{45}$$

where $\tilde{g} \equiv g' + s - \tilde{s} = g' + (1-\beta)s$. The covariance between the newly-defined score and non-score genetic components is

$$\begin{aligned}
\text{Cov}\,(\tilde{g}, \tilde{s}) &= \text{Cov}\,(g' + (1-\beta)s, \beta s) \\
&= \beta\text{Cov}\,(g', s) + \beta(1-\beta)\text{Var}\,(s) = \beta\rho r\sqrt{h^2 - r^2} + \beta(1-\beta)r^2 \\
&= \beta r\left(\rho\sqrt{h^2 - r^2} + (1-\beta)r\right) \\
&= \beta r\left(\rho\sqrt{h^2 - r^2} - \rho\sqrt{h^2 - r^2}\right) = 0.
\end{aligned} \tag{46}$$

Under the assumption of a jointly normal distribution, we have restored the independent component model of Eq. (3) using $\tilde{s}$ and $\tilde{g}$. The proportion of variance

in liability explained by the scaled PRS is $\mathrm{Var}(\tilde{s}) = \beta^2 r^2 = r^2 \left(1 + \frac{\rho\sqrt{h^2-r^2}}{r}\right)^2$. For $\rho > 0$, this will be greater than r^2, implying larger predicted risk reductions.

6.2 Shared environment

For some conditions, it is reasonable to assume that siblings (or other close relatives) share not only genetic risk factors, but also some of their environment. To address such cases, consider the following extension of the model of Eq. (4) Turley et al., 2021,

$$\begin{aligned} y_i &= (c + x_i) + (w + v_i) + (\epsilon_{\text{shared}} + \epsilon_{\text{unique},i}) \\ \epsilon_{\text{shared}} &\sim N\left(0, \pi\left(1 - h^2\right)\right) \\ \epsilon_{\text{unique},i} &\sim N\left(0, (1-\pi)\left(1 - h^2\right)\right). \end{aligned} \tag{47}$$

In the above equation, ϵ_{shared} is identical across embryos, while $\epsilon_{\text{unique},i}$ is independent between embryos. Thus, π is the proportion of the variance of the non-genetic ("environmental") risk factors that is shared between siblings.

As long as the disease status of family members of the embryos is unknown, the predicted risk reductions in Section 3 remain the same. This is because embryo selection is based on its PRS (the $c + x$ terms), and whether or not the non-genetic risk factors are shared does not change the risk of the selected embryo given its PRS.

Consider now the case when we condition on the known disease status of a sibling of the embryos (a previously born child). Define the liability of the embryo as y_{embryo} and the liability of the sibling (which is known to be greater than z_K in case the sibling is affected) as y_{sib}. We have

$$\begin{aligned} \mathrm{Cov}\left(y_{\text{embryo}}, y_{\text{sib}}\right) &= \mathrm{Cov}\left(x_{\text{embryo}} + v_{\text{embryo}} + \epsilon_{\text{unique;embryo}} + c + w + \epsilon_{\text{shared}},\right. \\ &\quad \left. x_{\text{sib}} + v_{\text{sib}} + \epsilon_{\text{unique;sib}} + c + w + \epsilon_{\text{shared}}\right) && (48) \\ &= \mathrm{Var}(c) + \mathrm{Var}(w) + \mathrm{Var}\left(\epsilon_{\text{shared}}\right) \\ &= \frac{r^2}{2} + \frac{h^2 - r^2}{2} + \pi(1 - h^2) \\ &= h^2\left(\frac{1}{2} - \pi\right) + \pi. && (49) \end{aligned}$$

In a model without shared environment, $\mathrm{Cov}\left(y_{\text{embryo}}, y_{\text{sib}}\right) = h^2/2$. Thus, the model with a shared environment can be mapped into the original model (Eq. (4)) by defining h'^2 such that

$$\begin{aligned} \frac{h'^2}{2} &= h^2\left(\frac{1}{2} - \pi\right) + \pi \\ h'^2 &= h^2(1 - 2\pi) + 2\pi. \end{aligned} \tag{50}$$

The mapping is valid as long as $h^2(1 - 2\pi) + 2\pi \leq 1$, or $\pi \leq 1/2$. We can then use the approach described in Section 4 for the estimation of the risk reductions,

by substituting h^2 with h'^2. This mapping is valid for any number of siblings with a known disease status.

To validate this result, we used simulations of the shared environment model of Eq. (47). Specifically, for a given set of embryos, we first sampled the three shared components, c, v, and ϵ_{shared}. We then sampled the unique components (including ϵ_{unique}) of each embryo and the born sibling. We rejected samples in which the sibling was unaffected (i.e., its liability was below the disease threshold). We then selected the embryo with the lowest PRS and computed the risk reduction. In comparison, we used our app to compute the risk reduction using the new heritability h'^2. The results (Figure 3) show good agreement between the two approaches. The same approach can be used when conditioning on the disease status of a single other relative, but not in other settings.

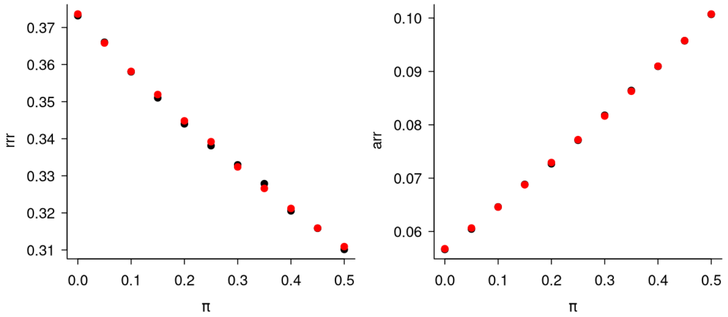

Figure 3: The relative and absolute risk reductions with a shared environment between siblings. We used $K = 0.1, r^2 = 0.1, h^2 = 0.3$, and $n = 5$, and conditioned on one affected sibling. The x-axis shows π, the proportion of non-genetic variance shared between siblings. The case $\pi = 0$ corresponds to the original model without shared environments. Red circles show the risk reduction in simulations of the model with a shared environment (one million repeats for each value of π). The black circles show the estimates using the original model but with adjusted heritability h'^2 (Eq. (50)), which agree well with the simulations. The relative risk reduction decreases with π and the absolute risk increases, which is expected given that having a shared environment with an affected sibling increases the baseline risk of the embryo (see Lencz et al., 2021).

6.3 Gene-environment correlation

Another possible deviation from our original model is correlation between the genetic and non-genetic (environmental) risk factors (Abdellaoui et al., 2022).

To account for such correlation, we modify the model of Eq. (3) as follows.

$$\begin{aligned} y &= s + g' + \epsilon \\ \mathrm{cor}\,(s, \epsilon) &= \rho \\ \mathrm{cor}\,(g', \epsilon) &= \rho. \end{aligned} \tag{51}$$

We assumed that the correlation is the same between the environment and either the score or the non-score genetic components. Given the dependence between the components of y, we adjust $\mathrm{Var}\,(\epsilon)$ to guarantee that $\mathrm{Var}\,(y) = 1$. To study the impact of the gene-environment correlation, we use simulations, considering here only the unconditional case. Specifically, we draw values of s_i, g'_i, e_i for the embryos from a multivariate normal distribution with the covariance matrix implied by Eq. (51) and with the constraint that $\mathrm{Cov}\,(s_i, s_j) = 1/2$ and $\mathrm{Cov}\left(g'_i, g'_j\right) = 1/2$ for sibling embryos. We then selected the embryo with the lowest PRS and computed the risk reductions.

We present the results in Figure 4. As expected, a positive gene-environment correlation implies larger risk reductions compared to the uncorrelated case, given that selecting for a low genetic score also selects for low non-genetic risk. The effect is opposite for a negative gene-environment correlation, where selecting for a low genetic score increases non-genetic risk and thus decreases the risk reductions.

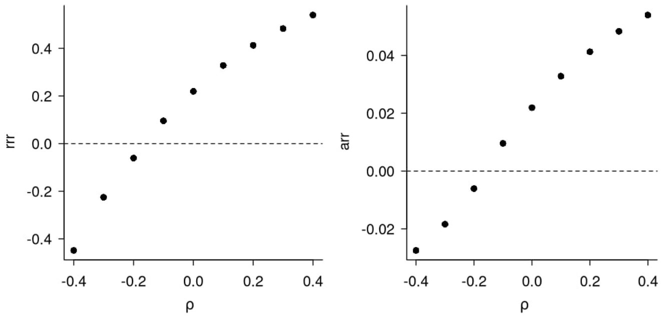

Figure 4: The risk reduction under gene-environment correlation. We set $K = 0.1, r^2 = 0.1, h^2 = 0.3$, and $n = 2$ and did not condition on the disease status of any family member. The x-axis is the gene-environment correlation ρ. Each dot corresponds to one million simulations from the model of Eq. (51). The left and right panels show the relative and absolute risk reductions, respectively.

7 Supplementary Tables

Table 1: Parameters used in the app for predefined diseases.
In Monti et al., 2024, the number are taken from their pgsCompaR package, looking at European population in the UK biobank. All numbers are rounded to the values that appear in corresponding sliders in the web app. K is the prevalence of the disease, r^2 is the variance of the PRS (or the proportion of variance in liability it explains), and h^2 is the heritability.

Disease	$K(\%)$	r^2	h^2
Alzheimer's Disease	11.1[26]	0.16[26]	0.58[4]
Asthma	17[21]	0.04[21]	0.54[9]
Atrial Fibrillation	7.4[21]	0.04[21]	0.6[22]
Breast Cancer (Female only)	13.86[26]	0.14[26]	0.31[11]
Coronary Artery Disease	8.3[21]	0.05[21]	0.49[11]
Depression (MDD)	15[23]	0.06[23]	0.4[11]
Glaucoma	7[26]	0.08[26]	0.37[11]
Hypertension	46.9[26]	0.16[26]	0.35[11]
Inflammatory bowel disease	1.4[20]	0.08[20]	0.75[7]
Multiple Sclerosis	0.69[17]	0.11[17]	0.5[6]
Osteoporosis	34[26]	0.07[26]	0.34[9]
Prostate Cancer (Male only)	12.55[26]	0.19[26]	0.57[11]
Psoriasis	2.9[26]	0.12[26]	0.57[9]
Rheumatoid Arthritis	0.2[20]	0.14[20]	0.53[11]
Schizophrenia	0.87[5]	0.07[19]	0.81[11]
Type 1 diabetes	0.15[25]	0.25[24]	0.83[27]
Type 2 diabetes	19.9[26]	0.21[26]	0.26[11]

References

[1] Donald B. Owen. "Tables for Computing Bivariate Normal Probabilities". In: *The Annals of Mathematical Statistics* 27.4 (Dec. 1956). Number: 4 Publisher: Institute of Mathematical Statistics, pp. 1075–1090. ISSN: 0003-4851, 2168-8990. DOI: 10.1214/aoms/1177728074. URL: https://projecteuclid.org/journals/annals-of-mathematical-statistics/volume-27/issue-4/Tables-for-Computing-Bivariate-Normal-Probabilities/10.1214/aoms/1177728074.full.

[2] D. S. Falconer. "The inheritance of liability to certain diseases, estimated from the incidence among relatives". In: *Annals of Human Genetics* 29.1 (1965), pp. 51–76. ISSN: 1469-1809. DOI: 10.1111/j.1469-1809.1965.tb00500.x. URL: https://onlinelibrary.wiley.com/doi/abs/10.1111/j.1469-1809.1965.tb00500.x.

[3] D. B. Owen. "A table of normal integrals". In: *Communications in Statistics - Simulation and Computation* 9.4 (Jan. 1, 1980). Number: 4 Publisher: Taylor & Francis, pp. 389–419. ISSN: 0361-0918. DOI: 10.1080/03610918008812164. URL: https://doi.org/10.1080/03610918008812164.

[4] Margaret Gatz et al. "Role of genes and environments for explaining Alzheimer disease". In: *Archives of General Psychiatry* 63.2 (Feb. 2006), pp. 168–174. ISSN: 0003-990X. DOI: 10.1001/archpsyc.63.2.168.

[5] Jonna Perälä et al. "Lifetime prevalence of psychotic and bipolar I disorders in a general population". In: *Archives of General Psychiatry* 64.1 (Jan. 2007), pp. 19–28. ISSN: 0003-990X. DOI: 10.1001/archpsyc.64.1.19.

[6] Corrado Fagnani et al. "Twin studies in multiple sclerosis: A meta-estimation of heritability and environmentality". In: *Multiple Sclerosis* 21.11 (Oct. 2015), pp. 1404–1413. ISSN: 1477-0970. DOI: 10.1177/1352458514564492.

[7] Hannah Gordon et al. "Heritability in Inflammatory Bowel Disease: From the First Twin Study to Genome-Wide Association Studies". In: *Inflammatory Bowel Diseases* 21.6 (June 2015), pp. 1428–1434. ISSN: 1078-0998. DOI: 10.1097/MIB.0000000000000393. URL: https://pmc.ncbi.nlm.nih.gov/articles/PMC4450891/.

[8] Yifang Li and Sujit K. Ghosh. "Efficient Sampling Methods for Truncated Multivariate Normal and Student-t Distributions Subject to Linear Inequality Constraints". In: *Journal of Statistical Theory and Practice* 9.4 (Oct. 2, 2015). Publisher: Taylor & Francis, pp. 712–732. ISSN: 1559-8608. DOI: 10.1080/15598608.2014.996690. URL: https://doi.org/10.1080/15598608.2014.996690.

[9] Tinca J. C. Polderman et al. "Meta-analysis of the heritability of human traits based on fifty years of twin studies". In: *Nature Genetics* 47.7 (July 2015), pp. 702–709. ISSN: 1546-1718. DOI: 10.1038/ng.3285. URL: https://www.nature.com/articles/ng.3285.

[10] Yulai Cong, Bo Chen, and Mingyuan Zhou. *Fast Simulation of Hyperplane-Truncated Multivariate Normal Distributions.* version: 2. Feb. 17, 2017. DOI: 10.48550/arXiv.1607.04751. arXiv: 1607.04751[stat]. URL: http://arxiv.org/abs/1607.04751.

[11] Fernanda C. G. Polubriaginof et al. "Disease heritability inferred from familial relationships reported in medical records". In: *Cell* 173.7 (June 14, 2018), 1692–1704.e11. ISSN: 0092-8674. DOI: 10.1016/j.cell.2018.04.032. URL: https://pmc.ncbi.nlm.nih.gov/articles/PMC6015747/.

[12] Naomi R Wray et al. "Complex Trait Prediction from Genome Data: Contrasting EBV in Livestock to PRS in Humans: Genomic Prediction". In: *Genetics* 211.4 (Apr. 1, 2019), pp. 1131–1141. ISSN: 1943-2631. DOI: 10.1534/genetics.119.301859. URL: https://doi.org/10.1534/genetics.119.301859.

[13] Todd Lencz et al. "Utility of polygenic embryo screening for disease depends on the selection strategy". In: *eLife* 10 (Oct. 12, 2021). Ed. by Y M Dennis Lo, Mone Zaidi, and Qiongshi Lu. Publisher: eLife Sciences Publications, Ltd, e64716. ISSN: 2050-084X. DOI: 10.7554/eLife.64716. URL: https://doi.org/10.7554/eLife.64716.

[14] Patrick Turley et al. "Problems with Using Polygenic Scores to Select Embryos". In: *New England Journal of Medicine* 385.1 (June 30, 2021). Publisher: Massachusetts Medical Society, pp. 78–86. ISSN: 0028-4793. DOI: 10.1056/NEJMsr2105065. URL: https://www.nejm.org/doi/full/10.1056/NEJMsr2105065.

[15] Abdel Abdellaoui et al. "Gene-environment correlations across geographic regions affect genome-wide association studies". In: *Nature Genetics* 54.9 (Sept. 2022), pp. 1345–1354. DOI: 10.1038/s41588-022-01158-0.

[16] Danilo Cimadomo et al. "Opening the black box: why do euploid blastocysts fail to implant? A systematic review and meta-analysis". In: *Human Reproduction Update* 29.5 (Oct. 1, 2023), pp. 570–633. ISSN: 1460-2369. DOI: 10.1093/humupd/dmad010. URL: https://doi.org/10.1093/humupd/dmad010.

[17] Hengameh Shams et al. "Polygenic risk score association with multiple sclerosis susceptibility and phenotype in Europeans". In: *Brain* 146.2 (Feb. 1, 2023), pp. 645–656. ISSN: 0006-8950. DOI: 10.1093/brain/awac092. URL: https://doi.org/10.1093/brain/awac092.

[18] Stefan Wilhelm and Manjunath B. G. *tmvtnorm: Truncated Multivariate Normal and Student t Distribution.* Version 1.6. Dec. 5, 2023. URL: https://cran.r-project.org/web/packages/tmvtnorm/index.html.

[19] Sophie E. Legge et al. "Genetic and Phenotypic Features of Schizophrenia in the UK Biobank". In: *JAMA Psychiatry* 81.7 (July 1, 2024), pp. 681–690. ISSN: 2168-622X. DOI: 10.1001/jamapsychiatry.2024.0200. URL: https://doi.org/10.1001/jamapsychiatry.2024.0200.

[20] Remo Monti et al. “Evaluation of polygenic scoring methods in five biobanks shows larger variation between biobanks than methods and finds benefits of ensemble learning”. In: *American Journal of Human Genetics* 111.7 (July 11, 2024), pp. 1431–1447. ISSN: 1537-6605. DOI: 10.1016/j.ajhg.2024.06.003.

[21] Buu Truong et al. “Integrative polygenic risk score improves the prediction accuracy of complex traits and diseases”. In: *Cell Genomics* 4.4 (Apr. 10, 2024). ISSN: 2666-979X. DOI: 10.1016/j.xgen.2024.100523. URL: https://www.cell.com/cell-genomics/abstract/S2666-979X(24)00065-X.

[22] Bengt Zöller et al. “Heritability of Atrial Fibrillation Among Swedish Adoptees”. In: *Circulation: Genomic and Precision Medicine* 17.3 (June 2024), e004563. DOI: 10.1161/CIRCGEN.124.004563. URL: https://www.ahajournals.org/doi/10.1161/CIRCGEN.124.004563.

[23] Mark J. Adams et al. “Trans-ancestry genome-wide study of depression identifies 697 associations implicating cell types and pharmacotherapies”. In: *Cell* 188.3 (2025), 640–652.e9. ISSN: 0092-8674. DOI: https://doi.org/10.1016/j.cell.2024.12.002. URL: https://www.sciencedirect.com/science/article/pii/S0092867424014156.

[24] Aaron J. Deutsch et al. “Development and Validation of a Type 1 Diabetes Multi-Ancestry Polygenic Score”. In: *Diabetes* 75.1 (Nov. 14, 2025), pp. 205–214. ISSN: 0012-1797. DOI: 10.2337/db25-0772. URL: https://doi.org/10.2337/db25-0772.

[25] Boshen Gong et al. “Global, regional, and national burden of type 1 diabetes in adolescents and young adults”. In: *Pediatric Research* 97.2 (Feb. 2025), pp. 568–576. ISSN: 1530-0447. DOI: 10.1038/s41390-024-03107-5. URL: https://www.nature.com/articles/s41390-024-03107-5.

[26] Spencer Moore et al. *Development and validation of polygenic scores for within-family prediction of disease risks.* Aug. 14, 2025. DOI: 10.1101/2025.08.06.25333145. URL: https://www.medrxiv.org/content/10.1101/2025.08.06.25333145v2.

[27] Yuxia Wei et al. “Stable heritability of type 1 diabetes in a Swedish Nationwide Cohort Study”. In: *Nature Communications* 16 (June 17, 2025), p. 5327. ISSN: 2041-1723. DOI: 10.1038/s41467-025-60813-2. URL: https://pmc.ncbi.nlm.nih.gov/articles/PMC12174315/.
